# Color-CADx: a deep learning approach for colorectal cancer classification through triple convolutional neural networks and discrete cosine transform

**DOI:** 10.1038/s41598-024-56820-w

**Published:** 2024-03-22

**Authors:** Maha Sharkas, Omneya Attallah

**Affiliations:** 1 Electronics and Communications Engineering Department, College of Engineering and Technology, Arab Academy for Science, Technology, and Maritime Transport, Alexandria, Egypt; 2 Wearables, Biosensing, and Biosignal Processing Laboratory, Arab Academy for Science, Technology and Maritime Transport, Alexandria, 21937 Egypt

**Keywords:** Colorectal cancer, Convolutional neural networks (CNNs), Deep feature extraction, Feature fusion, Transfer learning, Feature selection, Discrete cosine transform (DCT), Cancer, Medical research, Engineering

## Abstract

Colorectal cancer (CRC) exhibits a significant death rate that consistently impacts human lives worldwide. Histopathological examination is the standard method for CRC diagnosis. However, it is complicated, time-consuming, and subjective. Computer-aided diagnostic (CAD) systems using digital pathology can help pathologists diagnose CRC faster and more accurately than manual histopathology examinations. Deep learning algorithms especially convolutional neural networks (CNNs) are advocated for diagnosis of CRC. Nevertheless, most previous CAD systems obtained features from one CNN, these features are of huge dimension. Also, they relied on spatial information only to achieve classification. In this paper, a CAD system is proposed called “Color-CADx” for CRC recognition. Different CNNs namely ResNet50, DenseNet201, and AlexNet are used for end-to-end classification at different training–testing ratios. Moreover, features are extracted from these CNNs and reduced using discrete cosine transform (DCT). DCT is also utilized to acquire spectral representation. Afterward, it is used to further select a reduced set of deep features. Furthermore, DCT coefficients obtained in the previous step are concatenated and the analysis of variance (ANOVA) feature selection approach is applied to choose significant features. Finally, machine learning classifiers are employed for CRC classification. Two publicly available datasets were investigated which are the NCT-CRC-HE-100 K dataset and the Kather_texture_2016_image_tiles dataset. The highest achieved accuracy reached 99.3% for the NCT-CRC-HE-100 K dataset and 96.8% for the Kather_texture_2016_image_tiles dataset. DCT and ANOVA have successfully lowered feature dimensionality thus reducing complexity. Color-CADx has demonstrated efficacy in terms of accuracy, as its performance surpasses that of the most recent advancements.

## Introduction

Cancer is one of the main diseases that has affected human health and caused high mortality rates. Among cancer types, colorectal cancer (CRC) ranks as the third most prevalent reason for mortality worldwide, as indicated by worldwide cancer statistics^1^. According to the 2022 estimates from the American Cancer Society, there will be approximately 1.5 million new cases of CRC in the United States, resulting in around 53,000 deaths^2^. CRC manifests in the colon or rectum and is caused by uncontrolled cell proliferation resulting from genetic mutations. The primary factors contributing to CRC encompass older age, polyps, excessive consumption of processed food, being overweight, alcohol abuse, smoking, familial predisposition to colon cancer, and similar factors. Researchers and physicians face significant challenges when it comes to detecting CRC. The early detection of CRC to reduce mortality rates and improve survivability has been the subject of numerous scientific and research studies. Faecal occult blood and faecal immunochemical tests are used to assess stool samples, followed by colonoscopy, which is a high-quality procedure used to diagnose CRC and analyze its positive effects. Conducting physical inspections is typically hazardous as they fail to consider the existential discrepancy, thereby diminishing the whole efficacy of cancer scoring^3^.

Histopathological examination is a diagnostic technique employed for the identification of cancer. A microscope is employed for histopathological examination in order to ascertain the precise site and extent of the tumor^4–6^. Histopathology image analysis (HIA) is crucial for the clinical identification of CRC. It is crucial to classify various kinds of carcinomas, evaluate their aggressiveness, and identify tumor locations from whole slide images (WSIs) in order to properly identify and manage CRC^7^. The histopathological diagnosis is complex and relies on manual inspection, and the expertise of a medical expert, demands extensive examination time, and is influenced by the subjective decision of physicians^7–9^. Clinical decision-making may be aided by the implementation of artificial intelligence (AI)-based CRC detection systems, which could improve the accuracy of diagnosis and automate the process of inspection which will consequently decrease the dependability on the expert pathologist and decrease mortality rates^10,11^.

AI and its sub-branch deep learning (DL) have been used in medical image processing for cancer detection and classification^12–15^ and several other diseases^16–25^. Early detection of CRC can be facilitated by the development of a computer-assisted diagnosis (CAD) system, enabling timely intervention and appropriate medical care. This will enhance the effectiveness and precision of diagnosis, enabling prompt treatment for patients. The expansion of CAD can be achieved by developing image-processing algorithms and employing DL approaches. Furthermore, a CAD system aids in expediting decision-making processes and minimizes the time required for interpreting and evaluating health information. The main obstacle, however, lies in determining the methodology to extract significant features from histopathology images acquired from colon tissue. Although hand-crafted feature extraction methods are useful for characterizing tissue states, they lack reliability in generating distinctive feature vectors that facilitate classification^26^.

Convolutional neural networks (CNNs) which are a subset of the DL algorithms have made great successes in medical image processing^19,27–32^. It has several architectures and has been widely utilized for CRC detection and classification. The literature showed that most previous studies relied on a single CNN architecture. Even those studies that employed several CNN architectures used each one independently to perform classification. However, fusing deep features of multi-CNNs with different structures is more favored as it usually enhances classification performance. Furthermore, most of the current CADs depended on deep features of high dimension and did not employ a feature reduction step to lower their size, thus lowering the cost of classification. Additionally, most CNNs of previous CADs relied on spatial information to accomplish the detection and classification. However, merging spatial and spectral knowledge could boost the detection and classification procedures. Moreover, several present CADs perform only binary classification of WSI into cancerous versus non-cancerous. Nevertheless, determining the sub-class of CRC is important to deciding the treatment and observation plans. In addition, many existing CADs have typically utilized a training dataset consisting of tens to hundreds of WSIs that have been meticulously annotated by professional pathologists to identify disease regions^33^. However, accurately annotating WSIs is a demanding and time-consuming task due to their large size and the fact that tumor regions are typically scattered throughout the image, mixed with a significant amount of non-cancerous regions. This has made it challenging to develop DL models for HIA^34,35^.

To overcome the previously mentioned limitations, this study proposes a CAD system referred as to “Color-CADx” to classify multiple CRC subclasses. Color-CADx utilizes three CNN models of different architectures instead of one. It also fuses deep features of these CNNs.It does not depend only on spatial information included in images but also employs spectral demonstration to perform classification. Therefore, it adopts the discrete cosine transform (DCT) with zigzag scanning to combine deep features of the three CNNs and obtain spatial-spectral representation. DCT is further used to reduce the huge dimensions of the merged features. Furthermore, a feature selection approach is used to select significant features, thus reducing training complexity. Color-CADx performs the classification without requiring to specify the regions of disease and does not need any segmentation procedure. Color-CADx employs individual classifiers including support vector machine (SVM) and ensemble classifiers to accomplish classification.

The main contribution and originality of this research can be summarized as follows:

1. Evaluating the CNN accuracy at different training–testing ratios to overcome overfitting problems.
2. Employing three CNNs of distinct topologies rather than using one CNN of unique structure.
3. Extracting deep features from the three CNNs and fusing these features.
4. Relying on spectral knowledge as well as spatial information to perform classification instead of using only spatial information which is not the case in previous studies.
5. Applying the DCT with zigzag scanning to acquire spectral demonstration and reduce fused features obtained from the different CNNs.
6. Evaluating the classification performance for different feature lengths using analysis of variance (ANOVA) feature selection to select the optimum feature length that provides the best accuracy.
7. Color-CADx executes the classification process autonomously, eliminating the need to specify disease regions or execute any segmentation procedure.

The subsequent sections of the paper are organized as follows. Section "Related works" presents relevant research studies that developed CAD models for the diagnosis of CRC. Section "Material and methods" outlines the techniques utilized in the development of Color-CADx and provides a comprehensive explanation of each step. Subsequently, Sect. "Experiments settings" outlines the procedures employed to set up the experiments, including the specific hyperparameter values employed for the CNNs, as well as the performance metrics utilized to assess the effectiveness of Color-CADx. Following that, Sect. "Results" presents the findings and results, while Sect. "Discussion" provides a discussion of the main findings and an explanation of the limitations presented in Color-CADx. Section "Conclusion" contains the conclusion.

## Related works

The need for fast and objective analysis of histopathology slides initiated the use of digital pathology systems. Work includes three main categories which are detection, segmentation, and classification. This paper is concerned with classification. Previous work introduces two tracks, one is based on extracting hand-crafted features and feeding them to a classifier to obtain the result, and the other is based on DL methods.

### Handcrafted feature extraction-based methods

In the handcrafted feature extraction track, six types of texture descriptors to classify eight types of CRC were used by the authors in^8^. The texture descriptors are Local binary patterns (LBP), lower-order and higher-order histogram features, Gabor filters, Gray-level co-occurrence matrix (GLCM), Perception-like features, and Combined feature sets. The authors used a new dataset of 5,000 histological images of human CRC. The accuracy of multiclass CRC classification reached 87.4%. Furthermore, in^11^, various machine learning algorithms were used to classify CRC. Features were extracted from 3D images of three different color spaces which are RGB, HSV, and L*A*B colors spaces using GLCM. The authors used a training dataset of 3504 images and a testing dataset of 1496 images. They used five common machine learning algorithms, which are Support Vector Machine (SVM), K-Nearest Neighbor (KNN), Artificial Neural Network (ANN), Classification Decision Tree (CDT) and Quadratic Discriminant Analysis (QDA). The proposed methodology showed that it can detect CRC. This achieved accuracy resulted from combining texture features from all color space channels. QDA using RGB provided the best performance rate for the used machine learning models which was greater than 97% for the training and testing sets. These ML-based methods for recognizing CRC have four primary constraints. Initially, the process of extracting and selecting appropriate features is laborious, as it relies on a trial-and-error approach. Also, they are prone to error. Furthermore, previous studies have employed a diverse range of classifiers that possess numerous parameters. Choosing an efficient classifier is a difficult undertaking^6^. Finally, they usually produce low classification performance.

### Deep learning-based methods

Currently, DL-based systems are employed to autonomously extract superior features from the data being provided. It is an effective instrument for identifying a range of health issues. A CNN is a prevalent DL architecture. Several CNN models have been investigated for the identification and categorization of CRC. For example, Inception-v3 CNN architecture was used in the study^36^. The work in this study is based on WSIs which were taken from several hospitals and sources where China contributed 8554 patients, the United States provided 1077 patients, and Germany provided more than 111 slides. The average accuracy and AUC reached 98.06% (95% confidence interval [CI] (97.36–98.75%) and 98.83% (95% CI 98.15–99.51). Whereas, the authors of reference^37^ proposed a two-step procedure for CRC classification. In the first step, AlexNet which is a pre-trained deep CNN architecture is used for feature extraction. Then multiple machine learning classifiers are used to perform classification. The proposed method reaches an average accuracy of about 99.44% for the binary and multiclass image datasets of histopathology cancer images. The paper^6^ introduced a CNN called CRCCN-Net, which was designed to classify multi-class colorectal tissue histopathological images for the purpose of CRC classification. Four pre-existing CNNs, namely CRCCN-Net, Xception, InceptionResNetV2, DenseNet121, and VGG16, were individually trained on the NCT-CRC-HE-100K and colorectal histology datasets, respectively. Additionally, the two datasets were combined and subsequently utilized to train pre-existing models and CRCCN-Net to classify multi-class CRC. The suggested network achieved an accuracy of 93.50% on the colorectal histology dataset and 96.26% on the NCT-CRC-HE-100K dataset. With the combined dataset, the novel network achieved 99.21% accuracy.

The ResNet architecture, along with an attention module, was employed in^26^ to produce extensive feature maps to classify various tissues in HIs. Furthermore, neighborhood component analysis (NCA) effectively addresses the limitation of computational complexity. The selected features were inputted into an SVM classifier to train the model. The hybrid procedure was validated and tested using the CRC-5000 and NCT-CRC-HE-100 K datasets. The hybrid algorithm attains accuracy rates of 98.75% and 99.76% on CRC datasets. The study^38^ studies different hyperparameters tuning of VGG19 to classify CRC using WSI reaching an accuracy of 96.4%. on the other hand, the authors of^39^ employed a generative adversarial network (GAN) to generate synthetic data and used Inception for CRC classification reaching an accuracy of 89.54%. Whereas, the study^40^ introduced a dual-resolution DL-based framework, known as WDRNet, which stands for weakly supervised learning. Annotation was initially mitigated through the utilization of a CNN trained using weakly supervised multi-instance learning. Furthermore, a dual-stream network design was implemented to acquire comprehensive information at a scale of 5 and specific details at a scale of 20. The WDRNet model demonstrated a high level of accuracy in identifying tumour images, achieving an accuracy rate of 0.977 at the slide level and 0.953 at the patch level.

ResNet-18 and ResNet-50 were trained on colon gland images in^41^. The models were trained to classify CRC into two classes which are benign and malignant. The prototypes were tested on three varieties of testing data (20%, 25%, and 40% of whole datasets). ResNet-50 proved to provide the most reliable performance for accuracy, sensitivity, and specificity over ResNet-18 for the three kinds of testing data. The best performance value on 20% and 25% test sets achieved a classification accuracy of above 80%, a sensitivity of above 87%, and a specificity of above 83%. for the three test assortments. The authors in^42^ selected an optimizer and modified the parameters of the CNN models which improved the classification accuracy as they suggested. The well-trained DL methods were compared on two different histological image open datasets; the first comprised 5000 H&E images of CRC and the second was NCT-HE-100K data set which is composed of images comprising nine organizational categories with an external validation of 7180 images. The accuracy was close to 99% in an internal testing set and 94.3% in an external testing set. ResNet50 in this study resulted in an accuracy rate of 99.69% on the same internal testing set and 99.32% on the same external testing set which outperformed the data of VGG19. Moreover, ResNet50 achieved 94.86% accuracy for the eight classes of the Kather-texture-2016-image-5000 for comparison purposes.

The authors of reference^43^ introduced a self-supervised Deep Adaptive Regularised Clustering (DARC) framework for pre-training a neural network. DARC uses an iterative process to group the acquired representations and then uses these group assignments as pseudo-labels to train the network's parameters. The authors created an objective function that combines a network loss with a clustering loss using an adaptive regularisation function to improve the discriminative quality of representations. This function is updated dynamically during training to enhance the learning of feasible representations. On the other hand, the paper^44^ introduced a refined deep-learning model based on VGG16 for classifying image-level textures based on the CRC dataset. To reduce overfitting and significantly improve classification accuracy, it is essential to fine-tune the model, particularly when the training dataset is limited, thus the VGG-16 pre-trained model was fine-tuned. The study^45^ created a novel approach that integrates transfer learning and a ResNet50 CNN model to enhance the accuracy of classifying histopathology images of CRC. The experimental results showed exceptional performance with a training accuracy of 99.99% and a validation accuracy of 99.77%, achieving excellent results.

The study^46^ proposed an attention training mechanism embedded in a CNN for multiclass CRC classification. The NCT-CRC-100K dataset was utilized to validate the effectiveness of the suggested methodology, resulting in a classification accuracy of 99.77%. In^47^, the author introduced a DL method which is based on unsupervised feature extraction where a sub-region of a tissue image is quantized. A deep belief network of consecutive (RBMs) was used where the extracted sub-regions pixels are fed to and the activation values of the hidden units in the last RBM layer are defined as the deep features of this subregion. These deep features are then clustered to learn the quantization in an unsupervised way. A Nikon Coolscope Digital Microscope with a 20 × objective lens is used to collect the dataset giving an image resolution of 480 × 640. Images in this dataset are categorized into three classes: normal, low-grade cancer, and high-grade cancer. The dataset has 3236 images which are taken from 258 patients. The dataset is randomly divided into two groups to provide the training and testing sets. The training has 1644 images taken from 129 patients which were classified as 510 normal cases, 859 low-grade cancer cases, and 275 high-grade cancer cases. On the other hand, the remaining patients which comprise the test set have 1592 images divided into 491 normal cases, 844 low-grade cancer cases, and 257 high-grade cancer cases. The average accuracy reached 96%.

Similarly, the authors of the study^48^ introduced a novel attention mechanism called MCCBAM, which combines channel attention and spatial attention mechanisms. A framework named HCCANet was created using CNN and MCCBAM. The study utilized 630 histopathology images that underwent denoising with Gaussian filtering. Grad-CAM was employed to enhance the comprehensibility of HCCANet by visualizing regions of interest. The experimental findings demonstrate that the HCCANet model surpasses four cutting-edge DL models. In the study^49^, the authors compared handcrafted feature extraction methods with deep learning-based approaches. Four CNN architectures were assessed: ResNet-101, ResNeXt-50, Inception-v3, and DenseNet-161. They also suggested two Ensemble CNN methods: Mean-Ensemble-CNN and Neural Network-Ensemble-CNN. The experimental results demonstrate that the suggested methods surpassed the hand-crafted feature-based techniques and CNN architectures.

Prior research indicated that the majority of past studies depended on a singular CNN design. Even studies that utilized multiple CNN architectures employed each one separately for classification. Combining deep features from multiple CNNs with varying structures is typically preferred as it often improves classification accuracy. Most current CADs relied on deep features of high dimensions and did not use feature reduction to decrease their size, which would reduce the cost of classification. Moreover, the majority of CNNs in previous computer-aided diagnoses depended on spatial information for detecting and classifying CRC. Integrating spatial and spectral information could enhance the efficiency of detection and classification processes. In addition, many current CAD systems only conduct binary classification of whole slide images (WSI) as either cancerous or non-cancerous. Identifying the subtype of CRC is crucial for determining the appropriate treatment and monitoring strategies. Moreover, numerous current CAD systems have commonly employed a training dataset comprising tens to hundreds of whole slide images (WSIs) that have been carefully annotated by expert pathologists to detect areas of disease. Annotating whole slide images (WSIs) can be challenging and time-consuming because of their extensive size and the dispersed nature of tumour regions within the image, which are often intermingled with a substantial amount of non-cancerous areas. Developing deep learning models for health impact assessment has become difficult due to this.

This study suggests a CAD system called "Color-CADx" to classify various CRC subclasses, aiming to address the limitations mentioned earlier. Color-CADx employs three CNN models with distinct architectures instead of a single one. It also integrates deep features of these CNNs.The classification process relies not only on spatial information from images but also utilizes spectral information. The method utilizes the discrete cosine transform (DCT) with zigzag scanning to merge deep features from three CNNs and generate a spatial-spectral description. DCT is also utilized to decrease the large dimensions of the combined features. A feature selection approach is utilized to choose important features, thereby decreasing training complexity. Color-CADx classifies without the need for specifying disease regions or using segmentation procedures.

## Material and methods

### Datasets description

This research is applied to two datasets which are the NCT-CRC-HE-100 K dataset and the Kather_texture_2016_image_tiles which are two publicly available datasets for CRC cancer classification. Details of the used datasets are given below.

#### NCT-CRC-HE-100 K dataset

**Figure 1:**
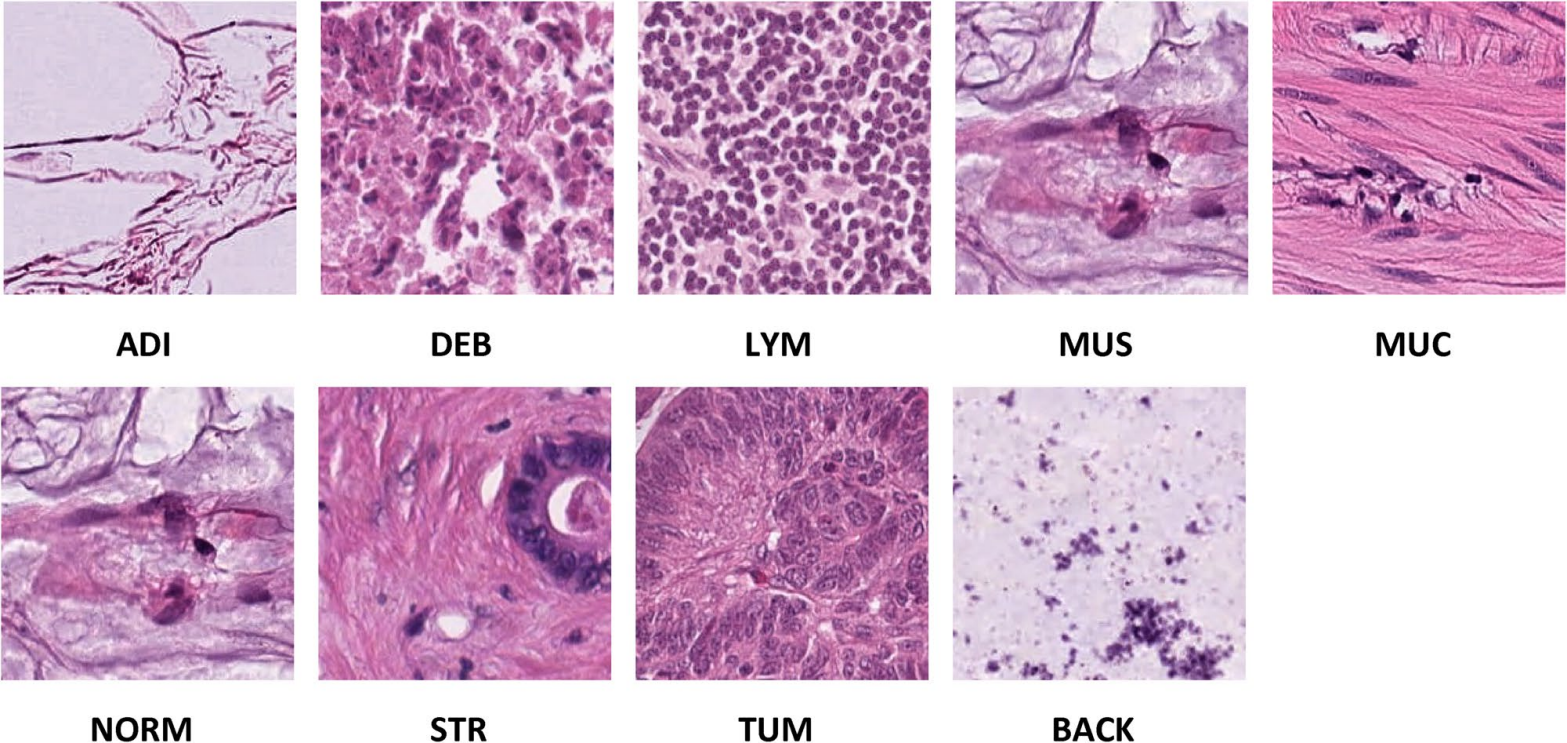
Samples from NCT-CRC-HE-100 K Dataset (20X Magnification factor).

The NCT-CRC-HE-100 K dataset publicly available in^50^ is a collection of distinct picture patches taken from histological images of human colorectal cancer (CRC) and healthy tissue stained with hematoxylin and eosin (H&E). All photos are 224 × 224 pixels and have a pixel size of 0.5 microns (MPP). All photos are color-normalized using Macenko's technique. The magnificator factor of the images in the dataset is 20×. Adipose (ADI), backdrop (BACK), debris (DEB), lymphocytes (LYM), mucus (MUC), smooth muscle (MUS), normal colon mucosa (NORM), cancer-associated stroma (STR), and colorectal adenocarcinoma epithelium (TUM) are the nine tissue types in the dataset. The National Center for Tumor Diseases in Heidelberg, Germany, and the UMM Pathology Archive provided the N = 86 H&E stained human cancer tissue slides from formalin-fixed paraffin-embedded (FFPE) samples for the dataset (University Medical Center Mannheim, Mannheim, Germany). The distribution of images among CRC classes is shown in Table 1. Samples from the nine classes of the dataset are shown in Fig. 1.

**Table 1:** The distribution of images among CRC classes for the NCT-CRC-HE-100K dataset.

Class lable	No. images
TUM	1000
NORM	1000
MUC	1000
DEB	1000
ADI	1000
STR	1000
MUS	1000
LYM	1000
BACK	1000
SUM	1000

#### Kather_texture_2016_image_tiles

**Figure 2:**
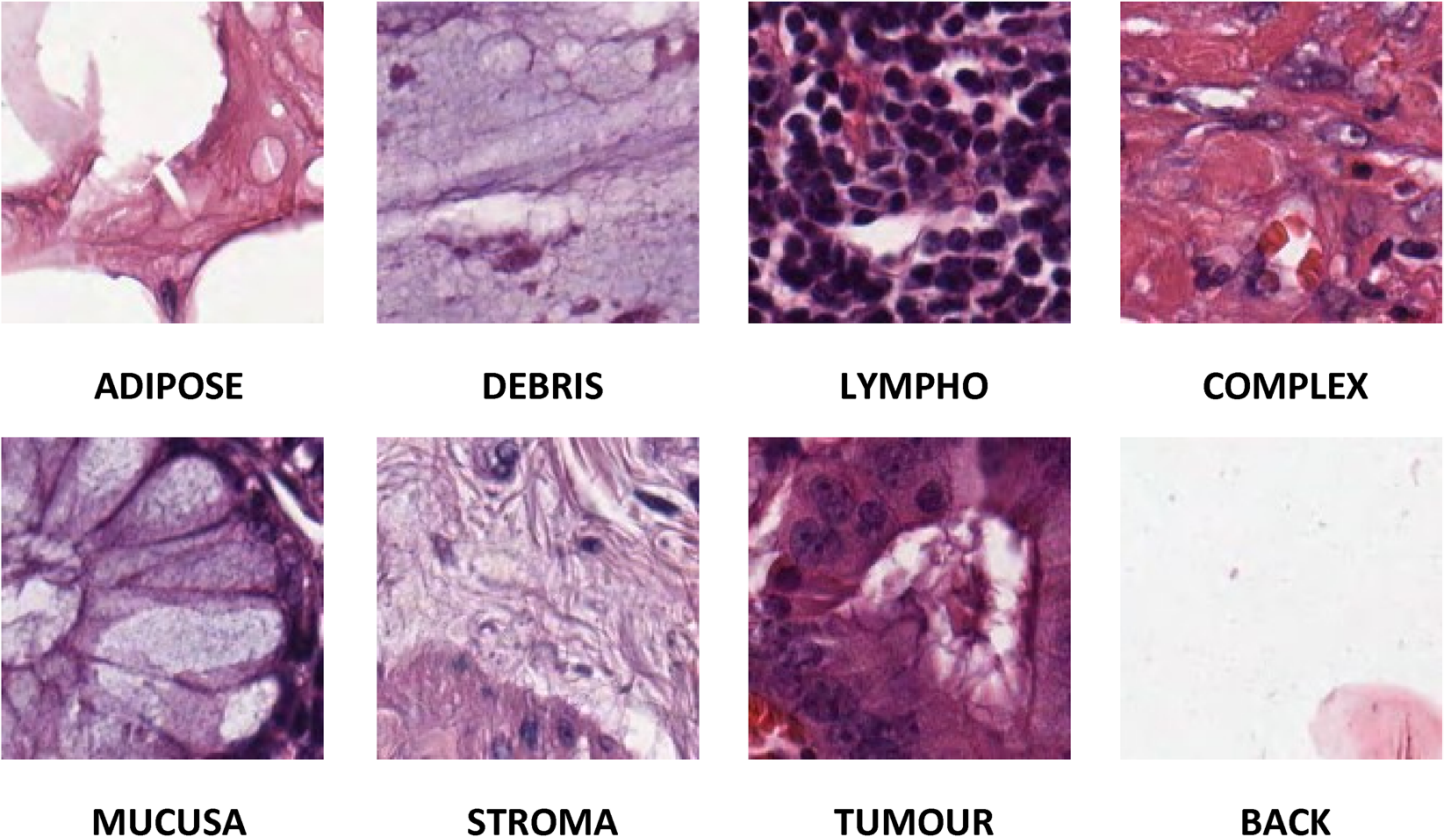
Samples from Kather_texture_2016_image_tiles Dataset (20X Magnificaton factor).

The University Medical Centre Mannhem in Germany provides a publicly accessible dataset known as Kather texture 2016^8,51^. The digitalized CRC tissue slides comprise samples derived from both low- and high-grade main tumors. The magnificator factor of the photos included in this dataset is 20 ×. Figure 2 illustrates eight distinct textures observed in tumor specimens: (1) cancerous epithelium (TUMOUR), (2) stromal cells (STROMA), (3) stromal tissue (COMPLEX), (4) immune cells (LYMPHO), (5) mucus and debris (DEBRIS), (6) glandular mucus (MUCOSA), (7) adipose tissue (ADIPOSE), and (8) background (BACK). The dataset contains 5000 image tiles, each measuring 150 × 150 pixels and 74 µm × 74 µm in size. The slides are significantly enhanced, providing a 20-fold improvement in clarity. Additionally, they are enriched with formalin and other histopathological markers, facilitating the pathologist's ability to diagnose them with ease. The picture labels have undergone evaluation by the Institute of Mannheim University of Medical Sciences in Germany. Table 2 provides the distribution of images in each CRC class. Samples from the nine classes of the dataset are shown in Fig. 2.

**Table 2:** The distribution of images among CRC classes for the Kather texture 2016 dataset.

Class lable	No. images
TUMOUR	625
STROMA	625
COMPLEX	625
DEBRIS	625
ADIPOSE	625
BACK	625
MUCOSA	625
LYMPHO	625

### Proposed color-CADx

**Figure 3:**
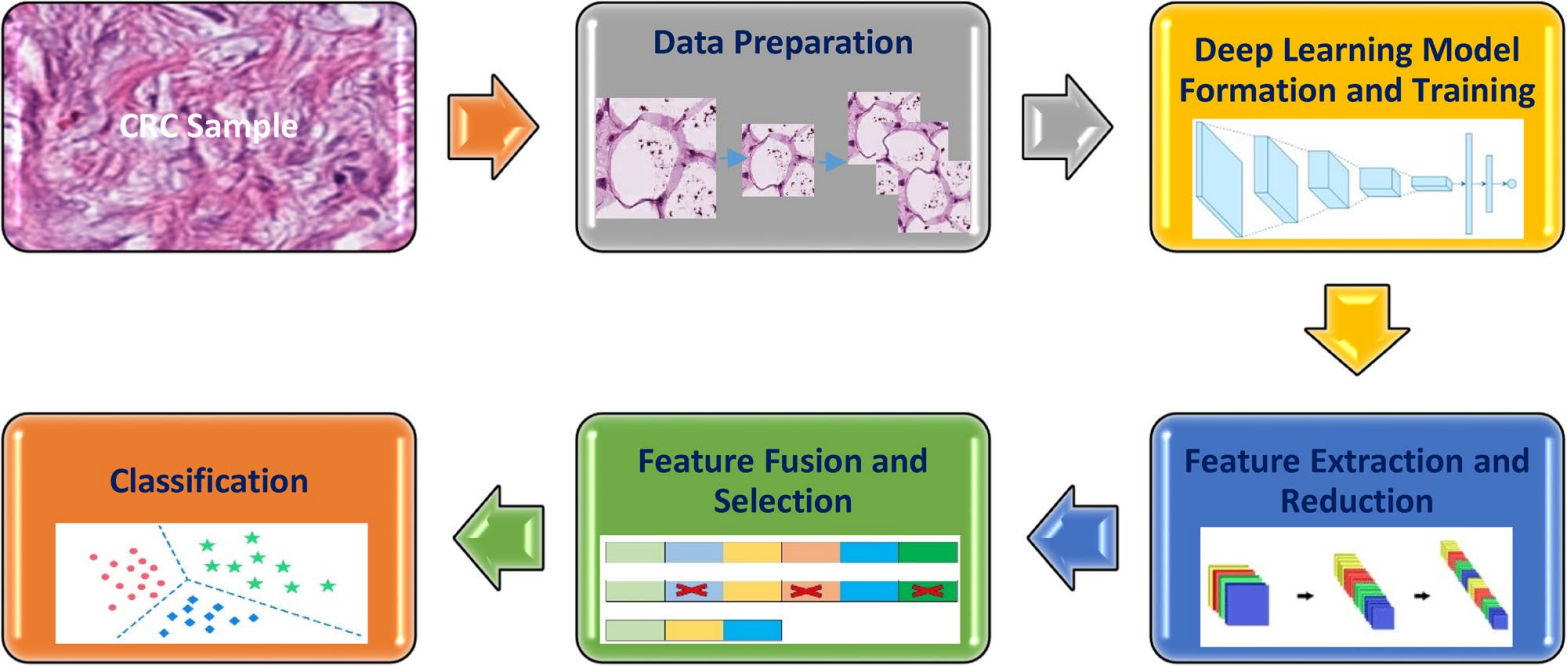
Summary of the steps of Color-CADx.

In order to accomplish CRC classification, Color-CADx implements several steps including data preparation, deep learning models formation and training, feature extraction and reduction, feature fusion and selection, and classification. In the data preparation step, each photo’s aspect is altered. Then, these images are split and augmented. Next, in the deep learning model formation and training, three pre-trained CNNs including AlexNet^52^, ResNet50^53^, and DenseNet201^54^ are constructed and then retrained using the two CRC datasets. After that in the feature extraction and fusion, deep features are extracted from each CNN and then their dimension is reduced using DCT. Afterward, these features are fused using DCT, and their dimensions are further diminished using a Zigzag scanning algorithm. In parallel, reduced DCT coefficients obtained from the three CNNs are concatenated and then a feature selection approach is used to select significant features. Lastly, individual and ensemble classifiers are employed to classify CRC images. The summary of Color-CADx steps is given in Fig. 3.

#### Data preparation

Initially, the aspects of the CRC images of both datasets are changed to be equivalent to the input length of each CNN. For ResNet50 and DenseNet201 they are modified to 224 × 224 × 3, whereas for AlexNet they are changed to 227 × 227 × 3. Next, two different training–testing ratios are employed to split data and ensure that there is no overfitting. The selected training–testing ratios are (70–30) and (60–40). After that, several augmentation techniques are used to increase the training photos to enhance training efficiency and further reduce overfitting. These augmentation techniques involve, flipping in vertical and horizontal directions, and rotation with the range (− 30, 30).

#### Deep learning model formation and training

Triple DL models are implemented utilizing transfer learning (TL) including AlexNet, ResNet50, and DenseNet201. AlexNet, despite being one of the earliest structures, continues to be utilized because of its satisfactory performance. This is due to its efficient computational ability and its outstanding performance with colour images^55^, as the datasets pointed out in this paper. AlexNet possesses a high learning rate and training pace, facilitating the learning process^56^ which is vital in medical applications requiring fast and precise diagnosis. It enhances network training efficiency without significantly increasing the workload and diminishes the reliance of gradients on the initial values and scale of parameters. The model's capacity to acquire hierarchical representations enables it to effectively capture complex patterns in medical images^56^. Besides, Integrating Local Response Normalisation (LRN) improves its ability to generalize, enabling it to detect minor differences and irregularities in medical data. On the other hand, ResNet is employed in this study as it is capable of convergence effectively with a reasonable computing expense even when the amount of layers is increased, unlike AlexNet CNN^57,58^. He et al.^58^ introduced a novel structure based on deep residual learning. This structure incorporates residuals, known as cutoffs, within the layers of a conventional CNN to intersect certain convolution layers simultaneously. These residuals enhance the efficiency of CNN. Furthermore, these residuals expedite and enhance the convergence process of the CNN despite the extensive number of deep convolution layers^59^. Besides, DenseNet 201 is utilized in this work since it uses dense connections between layers to decrease the number of parameters, enhance information flow, and promote feature reuse. This enhanced parameter efficiency results in a faster and more trainable network^60^.

TL is defined as the process of utilizing training data from a specific model to guide the development of a second model that is of a comparable nature. If the dataset in hand has an insufficient number of images and is used directly to train the CNN from scratch, it will not achieve good training performance. Thus, TL employs a CNN that has been pre-trained on a large dataset, like ImageNet, to perform a specific task. Subsequently, the pre-trained CNN model is applied to a novel dataset containing a reduced number of data samples, similar to the datasets utilized in our study^61^. In the medical domain, TL is frequently employed due to the scarcity of extensively annotated massive amounts of medical datasets comparable to the ImageNet database^62^. In this study, three pre-trained CNNs including AlexNet^52^, ResNet50^53^, and DenseNet201^54^ that were previously trained on the ImageNet dataset are constructed. TL is utilized to modify the output layer of convolutional CNNs to have 9 and 8 nodes, respectively, matching the number of categories in the NCT-CRC-HE-100 K dataset and the Kather_texture_2016_image_tiles dataset. The augmented pictures generated in the prior phase are subsequently employed to retrain the pre-trained CNNs by modifying certain hyperparameters of the CNNs, such as mini-batch size, learning rate, number of epochs, and validation rate. Further elaboration on the hyper-parameter finetuning will be provided in Sect. "Experiments settings".

#### Feature extraction and reduction

Once the retraining procedure of each CNN is terminated, TL is applied once again to obtain deep features from each CNN. For ResNet50 and DenseNet-201, features are acquired from the final average pooling layer whereas for ALexNet, features are obtained from the fully connected layer called “F7”. The dimensions of these features are 2048, 1920, and 4096 for ResNet50, DenseNet-201, and AlexNet. These features are of huge dimensions, thus a feature reduction step is required to lower their size. Therefore, DCT is adopted to diminish the dimension of each feature set independently. The DCT is a widely employed linear transformation technique in the field of signal analysis and processing. It facilitates the decomposition of the input data into its various frequency components^51^. Upon analyzing the input values utilizing DCT a matrix of DCT coefficients is produced. Only a subset of the components is retained while the remaining components are disregarded. The main advantage of DCT is that it possesses energy compaction property, indicating that important data information is concentrated in the lower frequency band. This property can be utilized for reducing feature dimensions. Most important features remain unchanged, ensuring the data quality is still preserved. This is valuable in scientific applications where balancing dimension reduction and quality maintenance is essential^63^. The attribute reduction process involves a critical step known as the selection of the DCT components. Usually, a conventional method such as zigzag is used to select from the DCT variables^53^. Therefore, in this study, DCT with zigzag scanning is used to diminish the size of features and obtain spectral information.

#### Feature fusion and selection

In this step, deep features of the three CNNs are combined using DCT. An ablation study is conducted to select the optimal number of features after DCT based on zigzag scanning. Feature selection (FS) is an important process that identifies the most beneficial variables in a given variable space and reduces their overall size, resulting in improved performance^64–66^. FS has the ability to disregard redundant or unrelated features within the collection of available features. Additionally, it expedites the training process and reduces training complexity. Furthermore, FS serves to mitigate overfitting during all stages of model training. Therefore, another approach for FS is investigated in this research. First, the DCT features acquired from the three CNNS in the previous step are concatenated. After that, analysis of variance (ANOVA) FS^67^ is performed to further reduce the dimension of the features thus lowering the complexity of the classification. ANOVA is a valid method for feature selection because it can detect significant differences between groups in a simple manner, identify important features for prediction rapidly, work with multiple classes and variables, assist in sensitivity analysis, prevent overfitting, remain computationally efficient, and offer results that are easy to interpret^68^. Another ablation study is conducted to show the accuracy versus features selected using ANOVA.

Balancing feature dimensionality reduction and retaining essential information is an essential issue in machine learning, especially for classification tasks. Dimensionality reduction techniques are used to decrease the number of features in a dataset to tackle problems such as computational complexity, and overfitting. Nevertheless, this decrease must be carefully monitored to guarantee that crucial information is preserved for precise classification. Excess reduction in dimensionality could result in the elimination of essential features that have discriminatory information, causing problems with the model's capacity to correctly categorize samples. Conversely, keeping an excessive number of features may add interference and result in the model becoming excessively intricate, thus impeding its ability to generalize to unfamiliar data. Choosing the correct balance requires the selection of suitable dimensionality reduction methods depending on the data's attributes. Parameter tuning is necessary to regulate the level of reduction and preserve the most pertinent information. It is crucial to use robust evaluation metrics to analyze the effect of dimensionality reduction on classification accuracy and find the best balance between simplifying the model and retaining important information. Achieving this equilibrium is essential for developing models that are computationally effective, generalize effectively to new data, and uphold outstanding precision in classification assignments^69,70^. Therefore, in this study ablation studies are conducted to examine the trade-off between the number of features and classification accuracy.

#### Classification

In order to perform the classification procedure of the CRC, the Color-CADx uses an individual classifier including Cubic SVM, and an ensemble classifier involving ensemble discriminate analysis (ESD). Color-CADx achieves the classification process through four experiments Experiment I investigates end-to-end classification using three CNNs: AlexNet, ResNet50, and DenseNet201. The purpose of this investigation is to address the issue of overfitting by utilizing two different training–testing ratios. The ratios for training and testing are 70–30 and 60–40, respectively. In the subsequent Experiment (II), deep features are extracted from every CNN and subsequently inputted into two shallow classifiers: the ESD and Cubic-SVM. Experiment III assesses the application of DCT for the purpose of reducing features. In Experiment IV, the features from various networks are combined using DCT, and then the zigzag scanning approach is applied to fused features. Then, selected features are fed to the same shallow classifiers. At the same time, the DCT features attained from the three CNNs in Experiment III are concatenated, and then ANOVA FS is adopted to select significant features, which are then used to feed the shallow classifiers.

## Experiments settings

### Validation metrics

The results of the proposed CAD framework are validated using several statistical validation metrics including F1-score, precision, accuracy, specificity (true positive rate (*TPR*)), and sensitivity (recall). Equations 1, 2, 3, 4 and 5 are used to compute these measures^12,18^

1 $$Accuracy=\frac{TP+TN}{TN+FP+FN+TP}$$

2 $$Sensitivity=\frac{TP}{TP+FN}$$

3 $$Specificity=\frac{TN}{TN+FP}$$

4 $$Precision=\frac{TP}{TP+FP}$$

5 $$F1-Score=\frac{2\times TP}{\left(2\times TP\right)+FP+FN}$$

where the total sum of CRC images that are well classified to the CRC class which they actually belong to is known as *TP, TN* is the sum of CRC images that do not belong to the CRC class intended to be classified, and truly do not belong to it. For each class of CRC, *FP* is the sum of all images that are classified as this CRC class but they do not truly belong to. For each class of CRC, *FN* is the entire sum of CRC images that are not classified as this CRC class.

### Hyperparameters finetuning

The hyper-parameters used are the minibatch size which is the amount of data included in each sub-epoch weight change and is chosen to be 10. Using small batch sizes usually achieves the best generalization performance. The learning rate determines the step size at each iteration while moving towards a minimum of a loss function. In my experiments, the learning rate was chosen to be 0.0001. The maximum number of epochs was chosen to be 20 as increasing the number of epochs did not improve the performance. The three used networks are trained with stochastic gradient descent with momentum techniques as this improves the rate of convergence and avoids getting trapped in a local minimum during convergence. Two distinct training–testing ratios are used to divide the data to prevent overfitting. The chosen training–testing ratios are 70–30 and 60–40. These splitting ratios were selected as they have been commonly used in the literature ^71–75^.

## Results

This research investigates several models for CRC classification which are evaluated using several experiments. Experiment I investigates end-to-end classification using three CNNs which are AlexNet, ResNet50, and DenseNet201 with two training–testing ratios to overcome overfitting. The training–testing ratios are 70–30 and 60–40. In Experiment II, features are extracted from each network and passed to two the SVM and ESD classifiers. Experiment III evaluates the use of the DCT for feature reduction. Next, in Experiment IV, features from different networks are fused using DCT, and zigzag scanning is used to select features and fed to the SVM and ESD classifiers. in addition, DCT features attained from each CNN are concatenated and then ANOVA FS is applied to select a significant number of lower features. This section will illustrate the results attained in each experiment.

### Experiment I results

In this section, the results of the end-to-end classification of the AlexNet, DenseNet201, and ResNet50 are shown. The accuracy results are given in Tables 3 and 4 for 70–30 and 60–40 ratios respectively for the Kather_texture_2016_image_tiles and the NCT-CRC-HE-100K datasets. As can be noted from Table 3, the accuracy achieved using AlexNet, DenseNet201, and ResNet50 for 70–30 split of the Kather_texture_2016_image_tiles dataset is 92.11%, 93.52%, and 93.38%. While for the 60–40 split of the same dataset, the accuracy reached 91.75%, 94.2%, and 92.2%. As concluded from Table 3, the DenseNet201 provided the best classification accuracy. On the other hand, Table 4 shows that the accuracy obtained using AlexNet, DenseNet201, and ResNet50 for a 70–30 split of the NCT-CRC-HE-100K dataset is 92.11%, 93.52%, and 93.38% respectively. For a 70–30 split of the same dataset, the accuracy achieved is 91.75%, 94.2%, and 92.2% respectively. According to the findings in Table 4, for the NCT-CRC-HE-100K, the ResNet50 and the DenseNet201 provided the best classification accuracies.

**Table 3:** Accuracy results for end-to-end classifications for the Kather_texture_2016_image_tiles.

Model	Accuracy (%)
*70–30 split ratio*	
AlexNet	92.11
ResNet50	93.38
DenseNet201	93.52
*60–40 split ratio*	
AlexNet	91.75
ResNet50	92.2
DenseNet201	94.2

**Table 4:** Accuracy results for end-to-end classifications for 70–30 and 60–40 ratios of the NCT-CRC-HE-100K dataset.

Model	Accuracy
*70–30 split ratio*	
AlexNet	94
ResNet50	97.33
DenseNet201	96
*60–40 split ratio*	
AlexNet	96.31
ResNet50	96.86
denseNet201	97.03

### Experiment II results

In the second experiment, deep features are extracted from the three CNNs and are fed to shallow classifiers including Cubic-SVM and ESD to obtain accuracy. The obtained results are given in Tables 5 and 6 for the NCT-CRC-HE-100K and Kather_texture_2016_image_tiles dataset using the two training–testing ratios. After several investigations, it was found that the SVM classifier with the Cubic SVM and the ESD classifier always provided the best results and they are the ones employed in the rest of the paper. Table 5 indicates that for the NCT-CRC-HE-100K dataset, the AlexNet, ResNet50, and DenseNet201 attain accuracies of (97.5%, 97.4%), (97.4%, 97.5%), and (99.4%, 98.8%) for SVM and ESD using 70–30 split ratio, whereas the same deep features reached accuracies of (97.7%, 97.6%), (97.8%,97.4%) and (99.4%,98.9%) for SVM and ESD using 60–40 split ratio.

**Table 5:** Accuracy results (%)for the used CNNs features for 70–30 and 60–40 ratios for the NCT-CRC-HE-100K dataset.

Model	Cubic SVM	Ensemble classifier
*70–30 split ratio*		
AlexNet	97.5	97.4
ResNet50	97.4	97.5
DenseNet201	99.4	98.8
*60–40 split ratio*		
AlexNet	97.8	97.6
ResNet50	97.8	97.4
DenseNet201	99.4	98.9

**Table 6:** Accuracy results (%) for the used CNNs features for 70–30 and 60–40 ratios for the kather_texture_2016_image_tiles dataset.

Model	Cubic SVM	Ensemble classifier
*70–30 split ratio*		
AlexNet	92.1	91.6
ResNet50	95	95
DenseNet201	96.9	95.9
*60–40 split ratio*		
AlexNet	92.3	91.1
ResNet50	94.8	95.1
DenseNet201	97.1	95.9

Table 6 shows the accuracies achieved by AlexNet, ResNet50, and DenseNet201 on the Kather_texture_2016_image_tiles dataset dataset. Using a 70–30 split ratio, SVM and ESD obtained accuracies of (92.1%, 91.6%), (95.0%, 95.0%), and (96.9%, 98.8%) respectively. On the other hand, using a 60–40 split ratio, the identical deep features accomplished accuracies of (92.3%, 91.1%), (94.8%, 95.1%), and (97.1%, 95.9%) respectively for SVM and ESD. The DenseNet201 provided the best classification accuracies with a maximum accuracy of 97.1% for the Kather_texture_2016_image_tiles dataset and an average of 97% for the Kather_texture_2016_image_tiles dataset. The Cubic SVM classifier performed better than the ensemble classifier.

### Experiment III results

This experiment presents the power of the DCT for feature reduction^76^. Features extracted from each CNN are fed into the DCT. The results obtained using the NCT-CRC-HE-100K and Kather_texture_2016_image_tiles datasets after reduction using DCT are shown in Tables 7 and 8 respectively. Table 7 demonstrates that the accuracies attained using the Cubic SVM and ESD classifiers for the 70–30 split ratio of the NCT-CRC-HE-100K dataset are 97.7% and 97.1% for AlexNet and ResNet50, and 99.2% and 98.9% for DenseNet201. While for the 60–40 split ratio, the accuracies are 97.8% and 97.2% for AlexNet, 97.7% and 97.2% for ResNet50, and 99.2% and 98.7% for DenseNet201. These accuracies are attained with 1500, 1200, and 1000 features for AlexNet, ResNer50, and DenseNet CNNs which are much lower than that used in Experiment II ( (4096, 2048, 1920 features for AlexNet, ResNer50, and DenseNet CNNs) with higher or almost the same accuracies attained in Experiment II (Table 5). The findings suggest that the spectral information obtained from DCT typically enhances performance.

**Table 7:** Accuracy results (%) after applying the DCT process on deep features of the three CNNs for 70–30 and 60–40 ratios for the NCT-CRC-HE-100K dataset.

Model	Cubic SVM	Ensemble classifier
*70–30 split ratio*		
AlexNet	97.7	97.1
ResNet50	97.7	97.1
DenseNet201	99.2	98.9
*60-40 split ratio*		
AlexNet	97.8	97.2
ResNet50	97.7	97.2
DenseNet201	99.2	98.7

**Table 8:** Accuracy results (%) after applying the DCT process on deep features of the three CNNs for 70–30 and 60–40 ratios for the Kather_texture_2016_image_tiles dataset.

Model	Cubic SVM	Ensemble classifier
*70–30 split ratio*		
AlexNet	93.8	93.4
ResNet50	95.2	94.2
DenseNet201	96.7	95.7
*60–40 split ratio*		
AlexNet	92.3	91.8
ResNet50	95.0	94.3
DenseNet201	96.8	95.4

The accuracy results obtained with the Cubic SVM and ESD classifiers for the 70–30 split ratio of the Kather_texture_2016_image_tiles dataset are presented in Table 8. Specifically, the accuracies for AlexNet and ResNet50 are 93.8%, 93.4%, and 95.2%, 94.2% for Cubic SVM and ESD classifiers, while DenseNet201 achieves 96.7% and 95.7% for the same classifiers. In contrast, AlexNet and ResNet50 achieve accuracies of 92.3%, 91.8%, and 95.0%, 94.3% for the 60–40 split ratio, whereas DenseNet201 achieves 96.8% and 95.4%. The accuracies achieved with 1500, 1200, and 1000 features for AlexNet, ResNet50, and DenseNet CNNs are significantly lower than those features obtained in Experiment II, where 4096, 2048, and 1920 features were used for AlexNet, ResNet50, and DenseNet CNNs. However, the accuracies achieved in Experiment II (Table 6) are higher except for ESD. The results indicate that the spectral information provided by DCT usually improves performance.

### Experiment IV results

**Figure 4:**
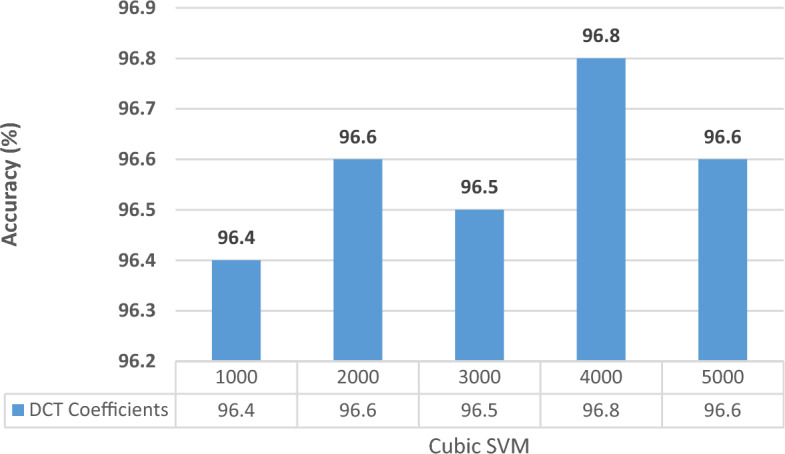
Classification accuracies for different DCT coefficients for the kather_texture_2016_image_tiles dataset with 60–40 training–testing ratios.

**Figure 5:**
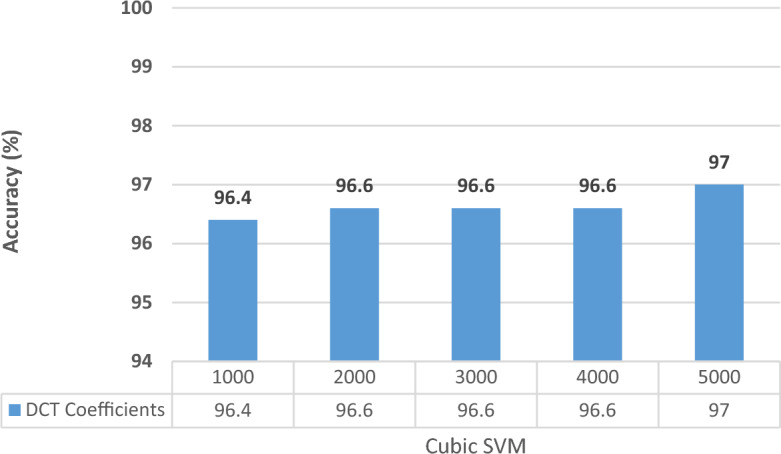
Classification accuracies for different DCT coefficients for the kather_texture_2016_image_tiles dataset with 70–30 training–testing ratios.

**Figure 6:**
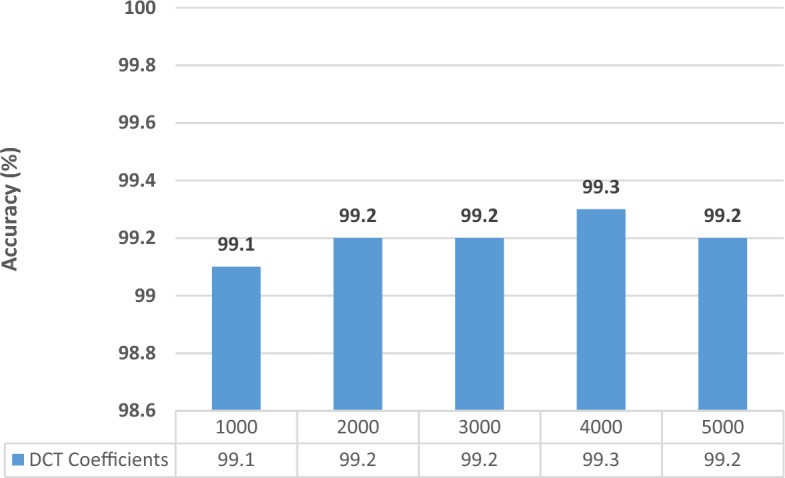
Classification accuracies for different DCT coefficients for the NCT-CRC-HE-100K dataset With 60–40 training–testing ratios.

**Figure 7:**
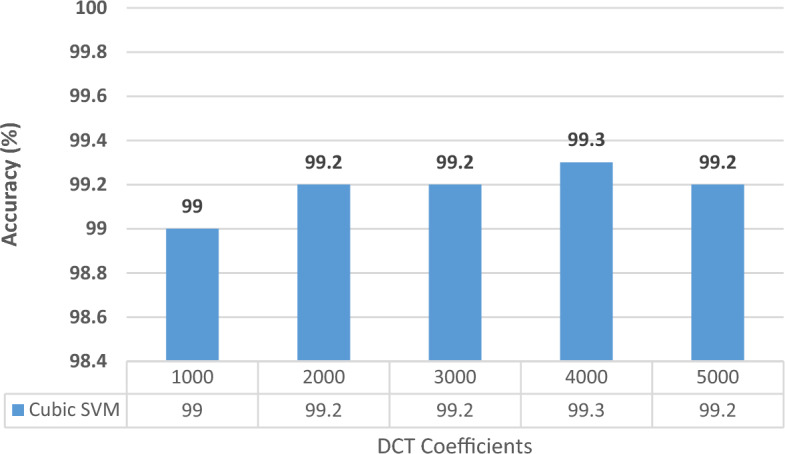
Classification accuracies for different DCT coefficients for the NCT-CRC-HE-100K dataset With 70–30 training–testing ratios.

In this experiment, all the extracted features from the three CNNs at the two selected training–testing ratios are fused using DCT. Different feature lengths are investigated to choose the optimum length that provides the best accuracy results. Note that, since Cubic SVM always attained higher performance than ESD in Experiments II and III for both datasets, it will be only used in Experiments IV and V. The results are given in charts provided in Figs. 4 and 5 for the Kather_texture_2016_image_tiles dataset and Figs. 6 and 7 for the NCT-CRC-HE-100K dataset for 70–30 and 60–40 split ratios. As shown in Figs. 4 and 5, for both split ratios of the Kather_texture_2016_image_tiles dataset. After fusing features using DCT, only 4000 and 5000 coefficients can provide the peak accuracy of 96.8% and 97.0% for the 70–30 and 60–40 splits respectively which are higher than those accuracies attained in Experiment III (Table 8). These results verify the DCT is capable of fusing features while reducing their dimension. Besides, the spatial-spectral information is superior to using just spatial representation. The feature dimensionality results in a decrease of almost 50% in the feature vector which results in reduced computational complexity.

Figures 6 and 7 illustrate the results obtained from the Kather_texture_2016_image_tiles dataset, considering both split ratios. By applying the Discrete Cosine Transform (DCT) to combine features, we found that only 4000 coefficients were necessary to achieve peak accuracy. Specifically, the accuracy reached 99.3% and 99.3% for the 70–30 and 60–40 splits, respectively. These accuracies are almost similar to that obtained in Experiment III (Table 7). These results confirm that the DCT can combine features while simultaneously decreasing their size. In addition, the spatial-spectral information surpasses the use of solely spatial representation. The reduction in feature dimensionality leads to a nearly 50% decrease in the size of the feature vector, resulting in a lower computational complexity.

**Figure 8:**
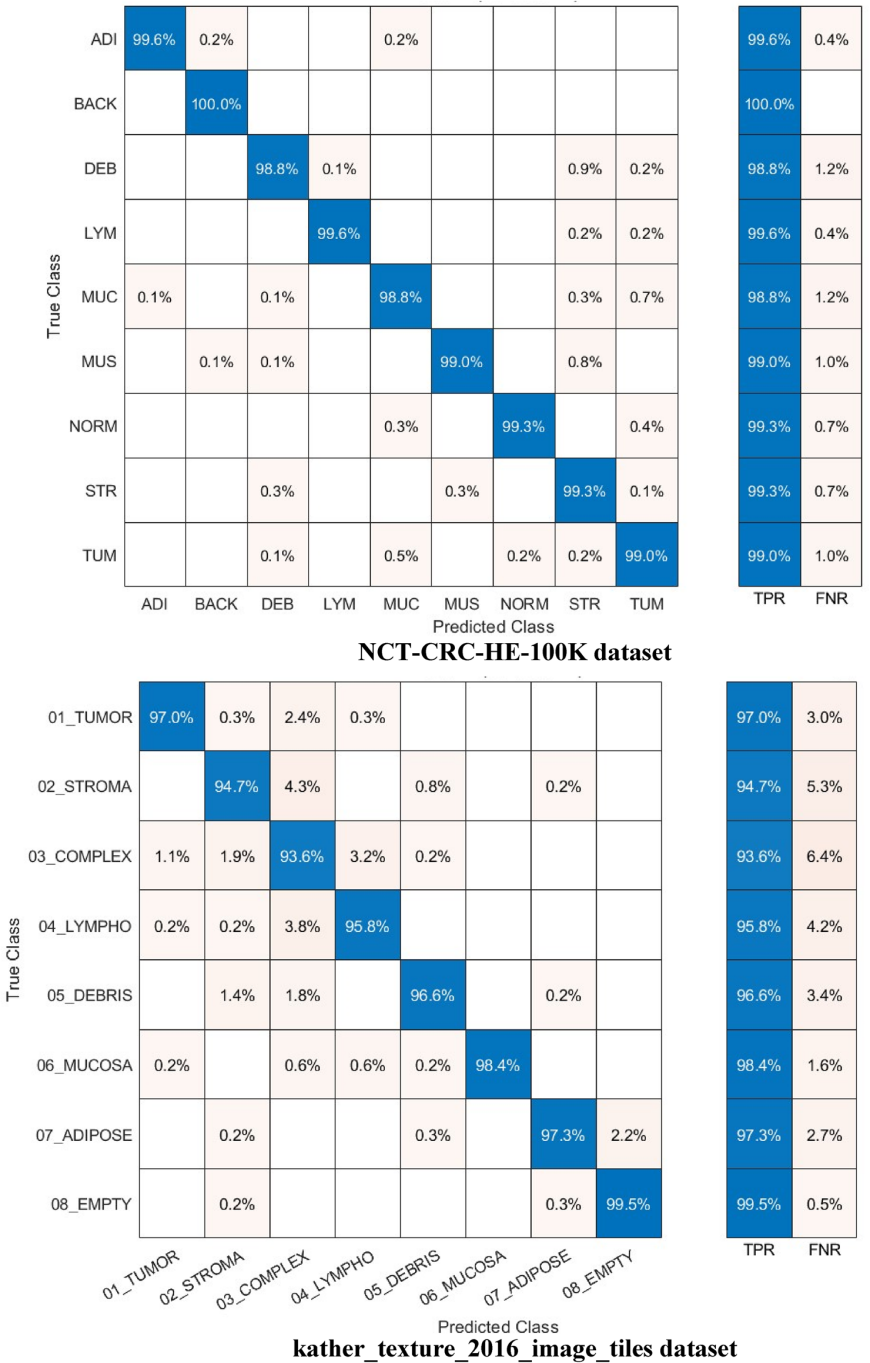
Confusion matrics realized with Cubic SVM trained with 4000 DCT components obtained via the kather_texture_2016_image_tiles dataset and the NCT-CRC-HE-100K dataset using the 70–30 split ratio.

**Figure 9:**
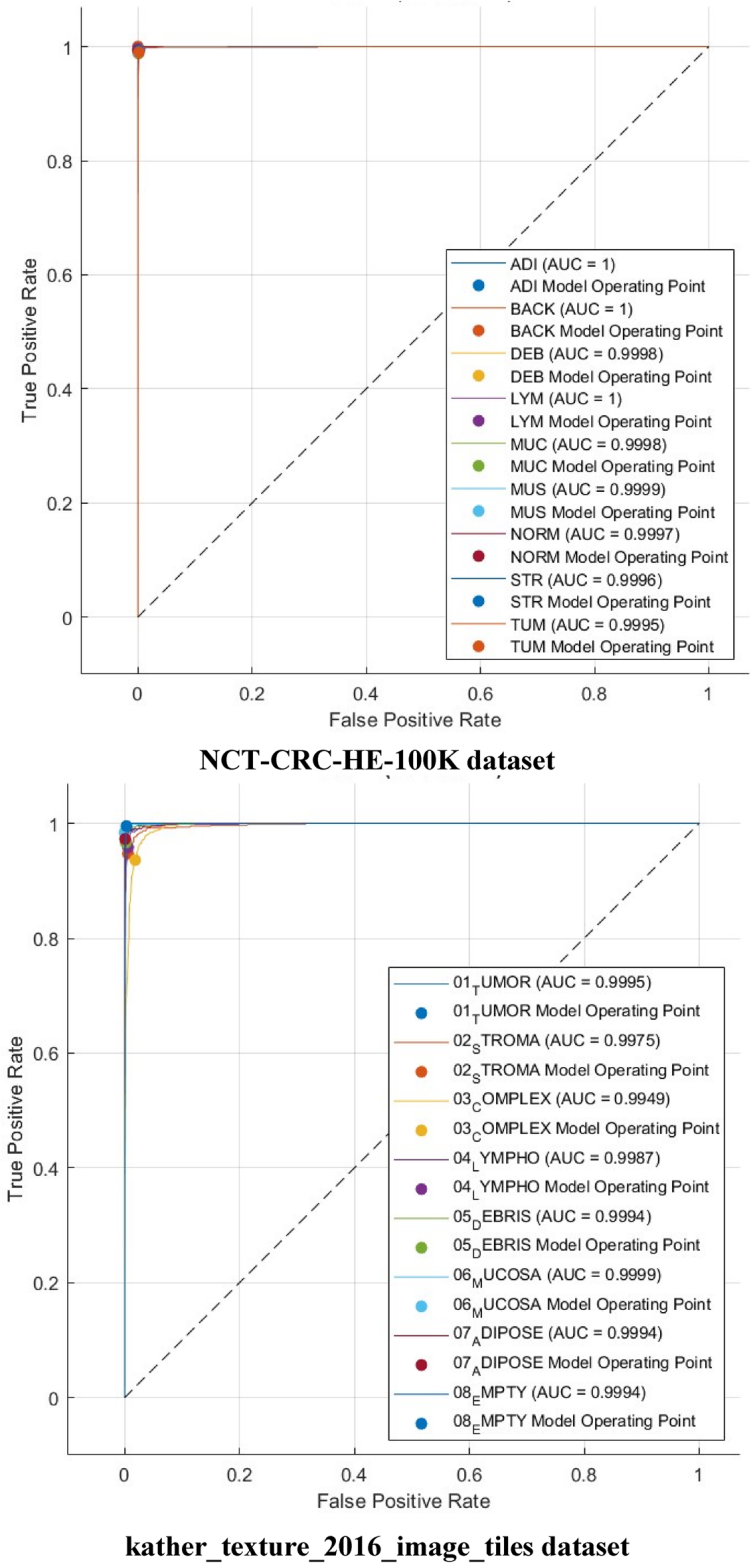
ROC curves realized with Cubic SVM trained with 4000 DCT components obtained via the kather_texture_2016_image_tiles dataset and the NCT-CRC-HE-100K dataset using the 70–30 split ratio.

Other performance metrics such as F1-score, precision, specificity, and sensitivity (recall) are calculated for the highest achieved accuracies in Experiment IV using the Cubic SVM and are given in Table 9 for the NCT-CRC-HE-100K dataset and the kather_texture_2016_image_tiles dataset. The mean and the standard deviation are calculated for the F1-score, precision, specificity, and sensitivity (recall) for all classes. Standard deviation is a measure of variation or dispersion between values in a set of data. The lower the standard deviation, the closer the data points tend to be to the mean (or expected value). On the other hand, a higher standard deviation indicates a wider range of values. The DenseNet201 always provided the best accuracies and its features are the ones used in Experiment IV. Also, the 70–30 training–testing ratio is the ratio used in Experiment IV as it attained higher performance than the 60–40 split ratio. Table 9 shows that the average precision, specificity, sensitivity, and F1-score using the 70–30 split ratio are 0.9672, 0.9952,0.9664, and 0.9667 for the kather_texture_2016_image_tiles dataset and 0.9924, 0.9990, 0.9923, and 0.9924 for the NCT-CRC-HE-100K dataset using 70–30 split ratio. Furthermore, the confusion matrices and receiving operating characteristics curve (ROC) for both datasets are determined and plotted in Figs. 8 and 9 respectively. Also, the area and ROC curve (AUC) is calculated.

**Table 9:** F1-score, precision, specificity, and sensitivity achieved with Cubic SVM trained with 4000 DCT components obtained via the kather_texture_2016_image_tiles dataset and the NCT-CRC-HE-100K dataset using 70–30 split ratio.

Value	Precision	Specificity	Sensitivity (recall)	F1-score
*kather_texture_2016_image_tiles dataset*				
Mean	0.9672	0.9952	0.9664	0.9667
Standard deviation	0.03545	0.00539	0.018965	0.025949
*NCT-CRC-HE-100K dataset*				
Mean	0.9924	0.9990	0.9923	0.9924
Standard deviation	0.00712	0.000912	0.00405	0.00488

Figure 8 illustrates that the Cubic SVM of the Color-CADx model accurately classifies each category in the NCT-CRC-HE-100 K dataset. The sensitivity rates for the ADI, BACK, DEB, LYM, MUC, MUS, NORM, STR, and TUM classes are 99.6%, 100%, 98.8%, 99.6%, 99.8%, 99.0%, 993.%, 99.3%, and 99.0%. In contrast, in the kather_texture_2016_image_tiles dataset, the Color-CADx model establishes a sensitivity of 97.0%, 94.7%, 93.6%, 95.8%, 96.6%, 98.4%, 97.3%, and 99.5% for TUMOR, STROMA, COMPLEX, LYMPHO, DEBRIS, MUCOSA, ADIPOSE, and EMPTY (BACKGROUND) categories. On the other hand, as shown in Fig. 9, the AUC for both datasets is either equal to 1 or almost 1.

Experiment IV also involves the concatenation of the DCT features acquired from the three CNNs, and then applying the ANOVA FS approach to select a reduced set of features. The results of the Cubic SVM classifier are shown in Table 10 for both datasets. Table 10 demonstrates that for the NCT-CRC-HE-100K dataset, the highest accuracy of 99.3% is achieved with 2000 features, whereas for the kather_texture_2016_image_tiles dataset, the maximum accuracy of 96.8% is obtained using 1000 features which is much lower than the 4000 features obtained when fusing the extracted features using DCT. Table 11 displays the following performance metrics for the kather_texture_2016_image_tiles dataset and the NCT-CRC-HE-100K dataset, using a 70–30 split ratio: average precision (0.9680 and 0.9932), specificity (0.9954 and 0.9991), sensitivity (0.9678 and 0.9931), and F1-score (0.9680 and 0.9931).

**Table 10:** Accuracy results (%) versus the number of features obtained with ANOVA FS.

# Features	1000	1500	2000	2500
*kather_texture_2016_image_tiles dataset*				
Cubic SVM	96.8	96.7	96.6	96.6
*NCT-CRC-HE-100K dataset*				
Cubic SVM	99.0	99.2	99.3	99.3

**Table 11:** F1-score, precision, specificity, and sensitivity achieved with Cubic SVM trained with 1000 and 2000 feat obtained via the kather_texture_2016_image_tiles dataset and the NCT-CRC-HE-100 K dataset using 70–30 split ratio.

Model	Precision	Specificity	Sensitivity (recall)	F1-score
*kather_texture_2016_image_tiles dataset*				
Cubic SVM	0.9680	0.9954	0.9678	0.9680
*NCT-CRC-HE-100K dataset*				
Cubic SVM	0.9932	0.9991	0.9931	0.9931

**Figure 10:**
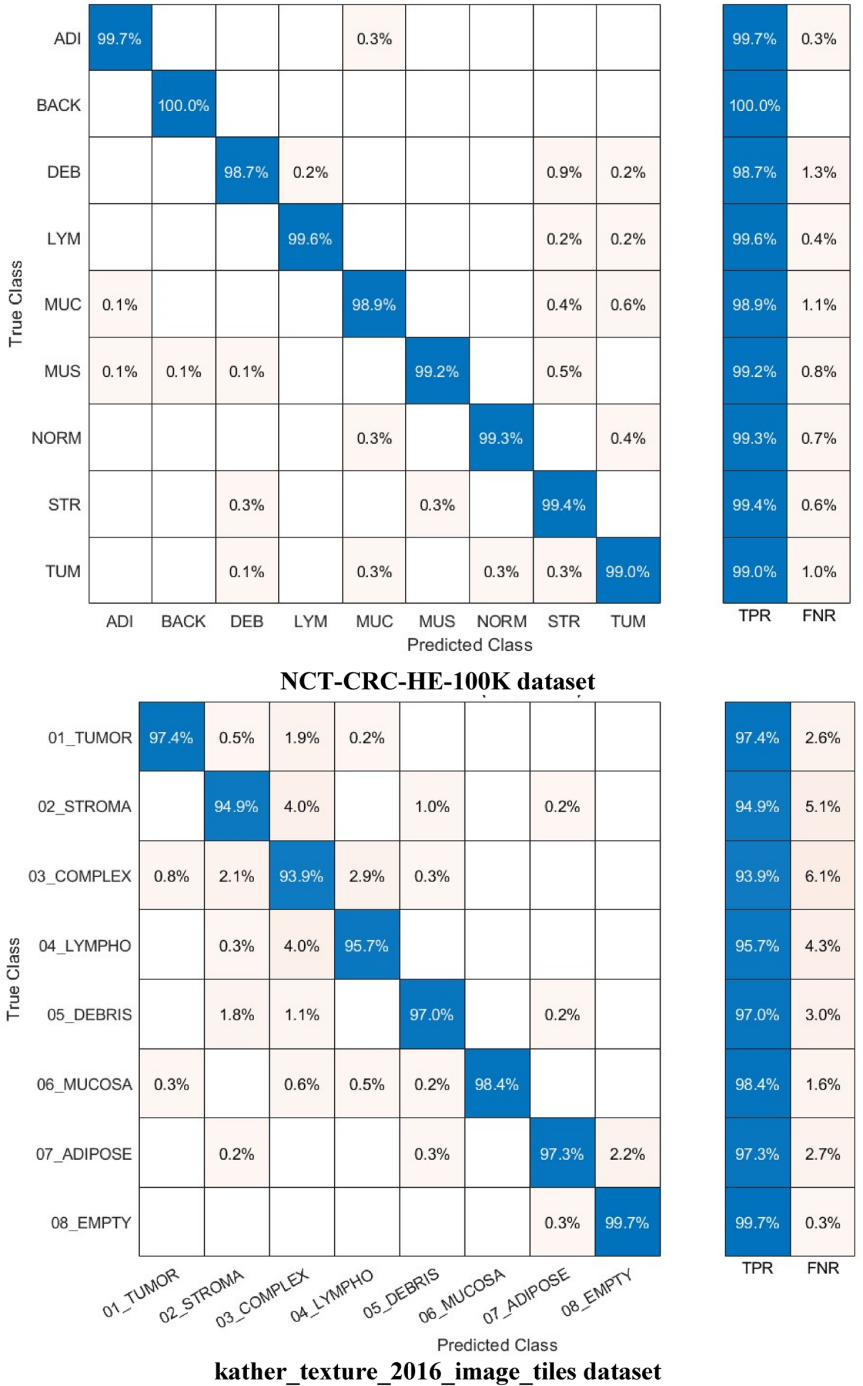
Confusion matrics realized with Cubic SVM trained with 1000 faetures obtained via the kather_texture_2016_image_tiles dataset and 2000 features acquired from the NCT-CRC-HE-100K dataset using the 70–30 split ratio.

**Figure 11:**
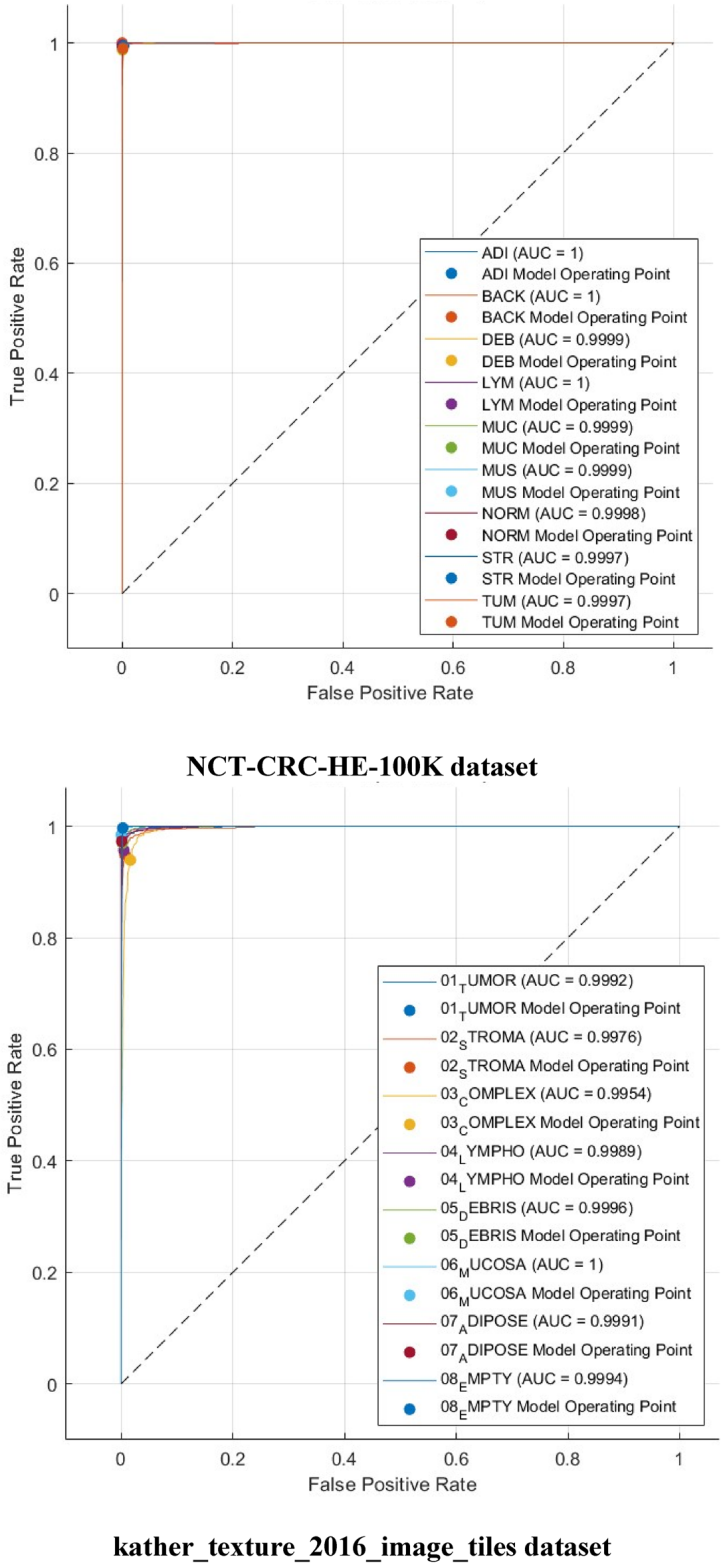
ROC curves realized with Cubic SVM trained with 1000 features obtained via the kather_texture_2016_image_tiles dataset and 2000 features acquired from the NCT-CRC-HE-100K dataset using the 70–30 split ratio.

The confusion matrices and ROC for both datasets for the best case achieved in Table 10 are calculated and graphed in Figs. 10 and 11, respectively (1000 features for the kather_texture_2016_image_tiles dataset and 2000 for NCT-CRC-HE-100 K dataset). In addition, the AUC is computed. The confusion matrices in Fig. 10 demonstrate that the Cubic SVM of the Color-CADx model effectively categorizes each class in the NCT-CRC-HE-100 K dataset. The sensitivity rates for the ADI, BACK, DEB, LYM, MUC, MUS, NORM, STR, and TUM classes are 99.7%, 100%, 98.7%, 99.6%, 99.9%, 99.2%, 99.4%, and 99.0% respectively. The kather_texture_2016_image_tiles dataset shows that the Color-CADx model achieves high sensitivity rates for various categories, including TUMOUR, STROMA, COMPLEX, LYMPHO, DEBRIS, MUCOSA, ADIPOSE, and EMPTY (BACKGROUND). Specifically, the sensitivity rates are 97.4%, 94.9%, 93.9%, 95.7%, 97.0%, 98.4%, 97.3%, and 99.7% respectively. However, as depicted in Fig. 9, the Area Under the Curve (AUC) for both datasets is either 1 or very close to 1.

## Discussion

This study aims to assess the effectiveness of using ensemble CNNs and transfer to automatically detect colorectal cancer WSIs. Besides, it seeks to investigate the capacity of DCT as a feature reduction and fusion algorithm. Therefore, in this study, a CAD system called "Color-CADx" is designed to accurately classify various subclasses of colorectal cancer (CRC). Color-CADx employs three convolutional neural network (CNN) models with distinct architectures, as opposed to using a single model. It additionally combines the deep features of these CNNs.The classification process does not solely rely on the spatial information provided by images but also utilizes spectral demonstration. Thus, it utilizes the discrete cosine transform (DCT) with zigzag scanning to merge the deep features from the three CNNs and achieve a spatial-spectral representation. The DCT is employed to decrease the vast dimensions of the combined features.

The classification process in Color-CADX is accomplished through four experiments. Experiment I examines the performance of three convolutional neural networks (CNNs)—AlexNet, ResNet50, and DenseNet201—in end-to-end classification. This investigation aims to tackle the problem of overfitting by employing two distinct training–testing ratios. The training and testing ratios are 70–30 and 60–40, respectively. In the following experiment (II), deep features are extracted from each CNN and fed into two shallow classifiers: the ESD and Cubic-SVM. Experiment III evaluates the utilization of DCT to decrease the number of features. The features obtained from multiple networks are aggregated utilizing DCT in Experiment IV, then followed by the zigzag scanning method to the merged features. Subsequently, the chosen features are inputted into the identical shallow classifiers. Furthermore, in Experiment IV, the DCT features obtained from the three CNNs are merged and then the ANOVA FS method is used to choose the most important features. These selected features are subsequently employed as input for the shallow classifiers.

Experiment II shows that extracting deep features using transfer learning is superior to end-to-end classification. This is obvious as the accuracy results obtained in Tables 5 and 6 (Experiment II) obtained with 4096, 2048, and 1920 features for ALexNet, ResNet50, and DenseNet201 are higher than those obtained in Tables 3 and 4 (Experiment I). In addition, Experiment III results prove that DCT was capable of decreasing the feature dimensionality to reach 1500, 1200, and 1000 features for ALexNet, ResNet50, and DenseNet201 with higher accuracy for AlexNet and ResNet and slightly lower accuracy in the case of DenseNet. On the other hand, the accuracies (99.3% and 96.8% for the NCT-CRC-HE-100 K and the kather_texture_2016_image_tiles datasets respectively) achieved in Experiment IV, when DCT was used to fuse features of the three CNNs, and then zigzag scanning was applied to select features verify that DCT is capable of enhancing the performance except for DenseNet which accomplished almost the same accuracy. Besides, the results also indicate that when concatenating DCT features attained from the three CNNs and applying ANOVA FS, the accuracy accomplished is 99.3% and 96.8% for the NCT-CRC-HE-100 K and the kather_texture_2016_image_tiles datasets with 2000 and 1000 features respectively.

### Comparisons with state-of-the-art methods

Table 11 presents a comparison of the results achieved by the proposed framework with the current cutting-edge methods for classifying CRC tissue. The suggested approach demonstrates superior performance compared to the majority of the previous works in the literature, with a substantial increase in accuracy compared to other studies. Furthermore, the findings of Color-CADx support the notion that the suggested study is an efficient and better method for detecting CRC from histopathological examination. The majority of the current approaches on the CRC datasets employ a solitary deep learning algorithm to classify the histopathological slide photos. Out of the approaches examined in Table 12, only studies^42^, and^77^ utilized multiple CNNs. however, the study^42^ used each CNN independently. Yet, their performance is lower than that achieved by the proposed method except for the accuracy achieved using the NCT-CRC-HE-100K employed in the study^42^.

**Table 12:** Comparison with the state-of-the-art methods based on the same datasets.

Article	Database	Method	Results
^42^	NCT-CRC-HE-100K kather_texture_2016_image_tiles dataset	5 different CNN Models	99.69% for NCT-CRC-HE-100K 94.86% for kather_texture_2016_image_tiles
^51^	NCT-CRC-HE-100K	VGG-16	98.7% for NCT-CRC-HE-100K
^6^	NCT-CRC-HE-100K kather_texture_2016_image_tiles dataset	CRCCN-Net	*NCT-CRC-HE-100K* Accuracy = 96.26% Sensitivity = 96.34% Precison = 96.44% Specificity = 99.52% F1-score = 96.38% *kather_texture_2016_image_tiles dataset* Accuracy = 93.5% Sensitivity = 93.62% Precison = 94.12% Specificity = 99.06% F1-score = 93.86%
^77^	NCT-CRC-HE-100K kather_texture_2016_image_tiles dataset	Ensemble learning driven on a CNN	*NCT-CRC-HE-100K* Accuracy = 96.16% Precision = 96.17% Sensitivity = 96.15% *kather_texture_2016_image_tiles dataset* Accuracy = 92.83% Precision = 92.83% Sensitivity = 93.11%
^26^	NCT-CRC-HE-100K kather_texture_2016_image_tiles dataset	ResNet + NCA + SVM	98.75% for kather_texture_2016_image_tiles 99.7% for NCT-CRC-HE-100K
^38^	NCT-CRC-HE-100K	VGG19	*NCT-CRC-HE-100K* Accuracy = 96.4% Precision = 94.22% Sensitivity = 94.44% F1-score = 94.44%
^39^	NCT-CRC-HE-100K	GAN + Inception	*NCT-CRC-HE-100K* Accuracy = 89.54% Precision = 86.84% Sensitivity = 86.62% F1-score = 98.70%
Color-CADx	NCT-CRC-HE-100K and kather_texture_2016_image_tiles dataset	ResNet + DesnseNet + AlexNet + DCT + SVM	*NCT-CRC-HE-100K* Accuracy = 99.3% Precision = 99.32% Specificity = 99.91% Sensitivity = 99.31% F1-score = 99.31% *kather_texture_2016_image_tiles* Accuracy = 99.3% Precision = 96.80% Specificity = 99.54% Sensitivity = 96.78% F1-score = 96.80%

### Limitations and forthcoming investigations

Notwithstanding these encouraging findings, this study is subject to several limitations. At first, there was a lack of research on optimizing strategies for selecting DL hyperparameters. Moreover, the Color-CADx neglected to consider the inherent uncertainty of the images. Besides, the study does not examine the presence of interference in medical pictures, which is considered one of the limitations of Color-CADx. Additionally, the research disregarded the utilization of other novel DL models, among them capsule networks and vision transformers. Although deep learning algorithms possess remarkable capabilities in examining medical photographs, the medical community maintains a high level of uncertainty regarding the legitimacy of CNNs given their opacity methodologies for making decisions. Due to the inherent inconsistency of CNNs in capturing attributes, it is challenging to discern their behavioral patterns and effectively harness any possible roadblocks. It is crucial to determine whether these profound features are associated with symptoms of disease and whether their results are supported by the knowledge of conventional medical specialists.

In the forthcoming project, the authors aim to enhance the performance of Color-CADx by incorporating additional lightweight CNN model designs, namely MobileNet^78^, EfficientNet^79^, and DarkNet19^80^. Moreover, explainable techniques such as Grad-CAM^81^ which have been used in medical images^82^ will be explored. In addition, the study will explore the application of filtering techniques to eliminate noise present in histopathological images^83^. Subsequent research will assess the effectiveness of the suggested method by comparing its performance to that of recent advanced DL models, including capsule networks^84^ and visual transformers^85^.

## Conclusion

CRC ranks third globally concerning both aggressiveness and mortality across different kinds of cancer. Timely and precise diagnosis of CRC is the most crucial stage in every malignancy. The study introduced Color-CADx as a method for automatically classifying colorectal tissue histopathological images. The work in this paper presented a new CAD model for CRC classification. The classification process of Color-CADx was conducted in four experiments. First in Experiment I, end-to-end classification was performed with three deep networks including AlexNet, ResNet50, and DenseNet. Features were then extracted using transfer learning from each network and passed to shallow classifiers for evaluation in Experiment II. DCT was then applied to the extracted features for feature reduction in Experiment III. Later, in Experiment IV, features from different classifiers were fused using DCT, and zigzag scanning was used to select features thus lowering the feature vector. In addition, DCT features acquired from each CNN were concatenated and then ANOVA FS was applied to these features to pick up a reduced set of features. Experiment II demonstrated that employing transfer learning to extract deep features outperformed end-to-end classification. The superiority of the accuracy results in Tables 5 and 6 (Experiment II) for ALexNet, ResNet50, and DenseNet201, achieved with 4096, 2048, and 1920 features, is evident when compared to the results in Tables 3 and 4 (Experiment I). Furthermore, the findings from Experiment III demonstrated that DCT successfully reduced the number of features to 1500, 1200, and 1000 for ALexNet, ResNet50, and DenseNet201, respectively. Notably, this reduction in feature dimensionality resulted in improved accuracy for AlexNet and ResNet, while the accuracy for DenseNet slightly decreased. However, in Experiment IV, when DCT was used to combine features from three CNNs and zigzag scanning was used to select features, accuracies of 99.3% and 96.8% were achieved for the NCT-CRC-HE-100K and kather_texture_2016_image_tiles datasets, respectively. This showed that DCT can improve performance, except for DenseNet which reached similar accuracy. In addition, the findings also explained that by combining DCT features obtained from the three CNNs and utilizing ANOVA FS, the achieved accuracy was 99.3% and 96.8% for the NCT-CRC-HE-100K and kather_texture_2016_image_tiles datasets, respectively. The number of features used was 2000 for the former dataset and 1000 for the latter. Color-CADx has proven efficacy in correctly categorizing CRC histopathological images. Therefore, it can serve as a valuable method for aiding medical professionals and technicians in accurately determining the particular kind of tissues in this examination. Consequently, cancerous specimens are less prone to going unnoticed, resulting in patients receiving appropriate and timely treatments with greater frequency.

## Data Availability

The NCT-CRC-HE-100 K dataset analyzed during the current study is available in the [Zenodo] repository, [https://zenodo.org/records/1214456]. The Kather_texture_2016_image_tiles dataset analyzed during the current study is available in the [Zenodo] repository, [https://zenodo.org/record/53169].

## References

[1] Sung H, Ferlay J, Siegel RL, Laversanne M, Soerjomataram I, Jemal A, Bray F (2021). Global cancer statistics 2020: GLOBOCAN estimates of incidence and mortality worldwide for 36 cancers in 185 countries. CA. Cancer J. Clin..

[2] Siegel RL, Miller KD, Jemal A (2019). Cancer statistics, 2019. CA Cancer J. Clin..

[3] Elmore JG, Longton GM, Carney PA, Geller BM, Onega T, Tosteson AN, Nelson HD, Pepe MS, Allison KH, Schnitt SJ (2015). Diagnostic concordance among pathologists interpreting breast biopsy specimens. Jama.

[4] Sengar, N., Mishra, N., Dutta, M.K., Prinosil, J., Burget, R. Grading of colorectal cancer using histology images. In *Proceedings of the 2016 39th International Conference on Telecommunications and Signal Processing* (TSP) 529–532 (IEEE, 2016).

[5] Alqudah A, Alqudah AM (2022). sliding window based support vector machine system for classification of breast cancer using histopathological microscopic images. IETE J. Res..

[6] Kumar A, Vishwakarma A, Bajaj V (2023). Crccn-Net: automated framework for classification of colorectal tissue using histopathological images. Biomed. Signal Process. Control.

[7] Zhou C, Jin Y, Chen Y, Huang S, Huang R, Wang Y, Zhao Y, Chen Y, Guo L, Liao J (2021). Histopathology classification and localization of colorectal cancer using global labels by weakly supervised deep learning. Comput. Med. Imaging Graph..

[8] Kather JN, Weis C-A, Bianconi F, Melchers SM, Schad LR, Gaiser T, Marx A, Zöllner FG (2016). Multi-class texture analysis in colorectal cancer histology. Sci. Rep..

[9] Attallah O, Anwar F, Ghanem NM, Ismail MA (2021). Histo-CADx: Duo cascaded fusion stages for breast cancer diagnosis from histopathological images. PeerJ Comput. Sci..

[10] Shafi ASM, Molla MMI, Jui JJ, Rahman MM (2020). Detection of colon cancer based on microarray dataset using machine learning as a feature selection and classification techniques. SN Appl. Sci..

[11] Alqudah AM, Alqudah A (2022). Improving machine learning recognition of colorectal cancer using 3D GLCM applied to different color spaces. Multimed. Tools Appl..

[12] Attallah O, Sharkas M (2021). Intelligent dermatologist tool for classifying multiple skin cancer subtypes by incorporating manifold radiomics features categories. Contrast Med. Mol. Imaging.

[13] Ragab DA, Attallah O, Sharkas M, Ren J, Marshall S (2021). A framework for breast cancer classification using multi-DCNNs. Comput. Biol. Med..

[14] Attallah O (1916). Cervical cancer diagnosis based on multi-domain features using deep learning enhanced by handcrafted descriptors. Appl. Sci..

[15] Bilal A, Shafiq M, Fang F, Waqar M, Ullah I, Ghadi YY, Long H, Zeng R (2022). IGWO-IVNet3: DL-based automatic diagnosis of lung nodules using an improved gray wolf optimization and InceptionNet-V3. Sensors.

[16] Masud M, Sikder N, Nahid A-A, Bairagi AK, AlZain MA (2021). A machine learning approach to diagnosing lung and colon cancer using a deep learning-based classification framework. Sensors.

[17] Tasnim Z, Chakraborty S, Shamrat FJM, Chowdhury AN, Nuha HA, Karim A, Zahir SB, Billah MM (2021). Deep learning predictive model for colon cancer patient using CNN-based classification. Int. J. Adv. Comput. Sci. Appl..

[18] Attallah O, Sharkas MA, Gadelkarim H (2019). fetal brain abnormality classification from MRI images of different gestational age. Brain Sci..

[19] Attallah O (2023). MonDiaL-CAD: monkeypox diagnosis via selected hybrid CNNs unified with feature selection and ensemble learning. Digit. Health.

[20] Attallah O (2022). An intelligent ECG-based tool for diagnosing COVID-19 via ensemble deep learning techniques. Biosensors.

[21] Attallah O (2022). ECG-BiCoNet: an ECG-based pipeline for COVID-19 diagnosis using bi-layers of deep features integration. Comput. Biol. Med..

[22] Attallah O, Sharkas MA, Gadelkarim H (2020). Deep learning techniques for automatic detection of embryonic neurodevelopmental disorders. Diagnostics.

[23] Bilal A, Imran A, Baig TI, Liu X, Long H, Alzahrani A, Shafiq M (2024). Improved support vector machine based on CNN-SVD for vision-threatening diabetic retinopathy detection and classification. Plos One.

[24] Bilal A, Liu X, Baig TI, Long H, Shafiq M (2023). EdgeSVDNet: 5G-enabled detection and classification of vision-threatening diabetic retinopathy in retinal fundus images. Electronics.

[25] Bilal A, Sun G, Mazhar S, Junjie Z (2021). Neuro-optimized numerical treatment of HIV infection model. Int. J. Biomath..

[26] Khazaee Fadafen M, Rezaee K (2023). Ensemble-based multi-tissue classification approach of colorectal cancer histology images using a novel hybrid deep learning framework. Sci. Rep..

[27] Giger ML, Doi K, MacMahon H (1988). Image feature analysis and computer-aided diagnosis in digital radiography. 3. automated detection of nodules in peripheral lung fields. Med. Phys..

[28] Kawata, Y., Niki, N., Ohmatsu, H., Kusumoto, M., Kakinuma, R., Mori, K., Nishiyama, H., Eguchi, K., Kaneko, M., Moriyama, N. Computer aided differential diagnosis of pulmonary nodules using curvature based analysis. In *Proceedings of the Proceedings 10th International Conference on Image Analysis and Processing* 470–475 (IEEE, 1999).

[29] Attallah O, Sharkas M (2021). GASTRO-CADx: A three stages framework for diagnosing gastrointestinal diseases. PeerJ Comput. Sci..

[30] Attallah O (2023). CerCan net: Cervical cancer classification model via multi-layer feature ensembles of lightweight CNNs and transfer learning. Expert Syst. Appl..

[31] Pacal I, Karaboga D, Basturk A, Akay B, Nalbantoglu U (2020). A comprehensive review of deep learning in colon cancer. Comput. Biol. Med..

[32] Attallah O, Samir A (2022). A wavelet-based deep learning pipeline for efficient COVID-19 diagnosis via CT slices. Appl. Soft Comput..

[33] Bejnordi BE, Veta M, Van Diest PJ, Van Ginneken B, Karssemeijer N, Litjens G, Van Der Laak JA, Hermsen M, Manson QF, Balkenhol M (2017). Diagnostic assessment of deep learning algorithms for detection of lymph node metastases in women with breast cancer. Jama.

[34] Pantanowitz L, Farahani N, Parwani A (2015). Whole slide imaging in pathology: Advantages, limitations, and emerging perspectives. Pathol. Lab. Med. Int..

[35] Attallah O (2021). MB-AI-His: Histopathological diagnosis of pediatric medulloblastoma and its subtypes via AI. Diagnostics.

[36] Wang KS, Yu G, Xu C, Meng XH, Zhou J, Zheng C, Deng Z, Shang L, Liu R, Su S (2021). Accurate diagnosis of colorectal cancer based on histopathology images using artificial intelligence. BMC Med..

[37] Fan J, Lee J, Lee Y (2021). A transfer learning architecture based on a support vector machine for histopathology image classification. Appl. Sci..

[38] Martínez-Fernandez E, Rojas-Valenzuela I, Valenzuela O, Rojas I (2023). Computer aided classifier of colorectal cancer on histopatological whole slide images analyzing deep learning architecture parameters. Appl. Sci..

[39] Jiang L, Huang S, Luo C, Zhang J, Chen W, Liu Z (2023). An improved multi-scale gradient generative adversarial network for enhancing classification of colorectal cancer histological images. Front. Oncol..

[40] Xu Y, Jiang L, Chen W, Huang S, Liu Z, Zhang J (2023). Computer-aided detection and prognosis of colorectal cancer on whole slide images using dual resolution deep learning. J. Cancer Res. Clin. Oncol..

[41] Sarwinda D, Paradisa RH, Bustamam A, Anggia P (2021). Deep learning in image classification using residual network (ResNet) variants for detection of colorectal cancer. Proced. Comput. Sci..

[42] Tsai M-J, Tao Y-H (2021). Deep learning techniques for the classification of colorectal cancer tissue. Electronics.

[43] Li J, Liu J, Yue H, Cheng J, Kuang H, Bai H, Wang Y, Wang J (2022). DARC: Deep adaptive regularized clustering for histopathological image classification. Med. Image Anal..

[44] Anju, T.E., Vimala, S. Finetuned-VGG16 CNN model for tissue classification of colorectal cancer. In *Intelligent Sustainable Systems. *Lecture Notes in Networks and Systems (eds Raj, J.S., Perikos, I., Balas, V.E.) 73–84 (Springer Nature Singapore, Singapore, 2023); Vol. 665, ISBN 978-981-9917-25-9.

[45] Peng, C.-C., Lee, B.-R. enhancing colorectal cancer histological image classification using transfer learning and ResNet50 CNN Model. In *Proceedings of the 2023 IEEE 5th Eurasia Conference on Biomedical Engineering, Healthcare and Sustainability (ECBIOS)* 36–40 (IEEE, 2023)

[46] Chen H, Li C, Li X, Rahaman MM, Hu W, Li Y, Liu W, Sun C, Sun H, Huang X (2022). IL-MCAM: An interactive learning and multi-channel attention mechanism-based weakly supervised colorectal histopathology image classification approach. Comput. Biol. Med..

[47] Sari CT, Gunduz-Demir C (2018). Unsupervised feature extraction via deep learning for histopathological classification of colon tissue images. IEEE Trans. Med. Imaging.

[48] Zhou P, Cao Y, Li M, Ma Y, Chen C, Gan X, Wu J, Lv X, Chen C (2022). HCCANet: Histopathological image grading of colorectal cancer using CNN based on multichannel fusion attention mechanism. Sci. Rep..

[49] Paladini E, Vantaggiato E, Bougourzi F, Distante C, Hadid A, Taleb-Ahmed A (2021). Two ensemble-CNN approaches for colorectal cancer tissue type classification. J. Imaging.

[50] Kather, J.N., Halama, N., Marx, A. NCT-CRC-HE-100K dataset: 100,000 histological images of human colorectal cancer and healthy tissue.

[51] Kather JN, Krisam J, Charoentong P, Luedde T, Herpel E, Weis C-A, Gaiser T, Marx A, Valous NA, Ferber D (2019). Predicting survival from colorectal cancer histology slides using deep learning: A retrospective multicenter study. PLoS Med..

[52] Krizhevsky, A., Sutskever, I., Hinton, G.E. Imagenet classification with deep convolutional neural networks. In *Proceedings of the Advances in Neural Information Processing Systems* 1097–1105 (2012).

[53] He, K., Zhang, X., Ren, S., Sun, J. Deep residual learning for image recognition. In *Proceedings of the Proceedings of the IEEE Conference on Computer Vision and Pattern Recognition* 770–778 (IEEE, 2016)

[54] Huang, G., Liu, Z., Van Der Maaten, L., Weinberger, K.Q. Densely connected convolutional networks. In *Proceedings of the Proceedings of the IEEE Conference on Computer Vision and Pattern Recognition* 4700–4708 (IEEE, 2017).

[55] Wang R, Xu J, Han TX (2019). Object instance detection with pruned alexnet and extended training data. Signal Process. Image Commun..

[56] Xu Y, Wang Y, Razmjooy N (2022). Lung cancer diagnosis in ct images based on alexnet optimized by modified bowerbird optimization algorithm. Biomed. Signal Process. Control.

[57] Talo M, Baloglu UB, Yıldırım Ö, Acharya UR (2019). Application of deep transfer learning for automated brain abnormality classification using MR images. Cogn. Syst. Res..

[58] He, K., Zhang, X., Ren, S., Sun, J. Deep residual learning for image recognition. In *Proceedings of the IEEE Conference on Computer Vision and Pattern Recognition* (2016).

[59] Attallah O (2021). CoMB-deep: Composite deep learning-based pipeline for classifying childhood medulloblastoma and its classes. Front. Neuroinformatics.

[60] Wakili MA, Shehu HA, Sharif MH, Sharif MHU, Umar A, Kusetogullari H, Ince IF, Uyaver S (2022). Classification of breast cancer histopathological images using DenseNet and transfer learning. Comput. Intell. Neurosci..

[61] Huh, M., Agrawal, P., Efros, A.A. What makes ImageNet good for transfer learning? *ArXiv Prepr. ArXiv160808614* (2016).

[62] Matsoukas, C., Haslum, J.F., Sorkhei, M., Söderberg, M., Smith, K. What makes transfer learning work for medical images: Feature reuse & other factors. In *Proceedings of the Proceedings of the IEEE/CVF Conference on Computer Vision and Pattern Recognition* 9225–9234 (2022).

[63] Kumar S, Mukherjee S, Pal AK (2023). An improved reduced feature-based copy-move forgery detection technique. Multimed. Tools Appl..

[64] Cai J, Luo J, Wang S, Yang S (2018). Feature selection in machine learning: A new perspective. Neurocomputing.

[65] Chizi, B.; Rokach, L.; Maimon, O. A survey of feature selection techniques. In *Encyclopedia of data warehousing and mining*, Second Edition. 1888–1895 (IGI Global, 2009).

[66] Attallah O, Karthikesalingam A, Holt PJ, Thompson MM, Sayers R, Bown MJ, Choke EC, Ma X (2017). Feature selection through validation and un-censoring of endovascular repair survival data for predicting the risk of re-intervention. BMC Med. Inform. Decis. Mak..

[67] Kemp, F. The handbook of parametric and nonparametric statistical procedures (2003).

[68] Nasiri H, Alavi SA (2022). A novel framework based on deep learning and ANOVA feature selection method for diagnosis of COVID-19 cases from chest X-ray images. Comput. Intell. Neurosci..

[69] Jia W, Sun M, Lian J, Hou S (2022). Feature dimensionality reduction: A review. Complex Intell. Syst..

[70] Othman, A.A., Hasan, T.M., Hasoon, S.O. Impact of dimensionality reduction on the accuracy of data classification. In *Proceedings of the 2020 3rd international conference on engineering technology and its applications (IICETA)* 128–133 (IEEE, 2020)

[71] Amin MS, Ahn H (2023). FabNet: A features agglomeration-based convolutional neural network for multiscale breast cancer histopathology images classification. Cancers.

[72] Tripathi, A., Kumar, K., Misra, A., Chaurasia, B.K. Colon cancer tissue classification using ML. In *Proceedings of the 2023 6th International Conference on Information Systems and Computer Networks (ISCON)* 1–6 (IEEE, 2023)

[73] Tripathi A, Misra A, Kumar K, Chaurasia BK (2023). Optimized machine learning for classifying colorectal tissues. SN Comput. Sci..

[74] Hamed EA, Tolba M, Badr N, Salem MA-M (2023). Large-scale histopathological colon cancer annotation model using machine learning techniques. Int. J. Intell. Comput. Inf. Sci..

[75] Alladi SM, Ravi V, Murthy US (2008). Colon cancer prediction with genetic profiles using intelligent techniques. Bioinformation.

[76] Pan, Z., Adams, R., Bolouri, H. Image recognition using discrete cosine transforms as dimensionality reduction. In *Proceedings of the IEEE-EURASIP Workshop on Nonlinear Signal and Image Processing; Citeseer* (2001).

[77] Ghosh S, Bandyopadhyay A, Sahay S, Ghosh R, Kundu I, Santosh KC (2021). Colorectal histology tumor detection using ensemble deep neural network. Eng. Appl. Artif. Intell..

[78] Sandler, M., Howard, A., Zhu, M., Zhmoginov, A., Chen, L.-C. Mobilenetv2: Inverted residuals and linear bottlenecks. In *Proceedings of the Proceedings of the IEEE Conference on Computer Vision and Pattern Recognition* 4510–4520 (2018).

[79] Tan, M., Le, Q. Efficientnet: Rethinking model scaling for convolutional neural networks. In *Proceedings of the International Conference on Machine Learning* 6105–6114 (PMLR, 2019).

[80] Redmon, J. Darknet: Open source neural networks in c (2013).

[81] Selvaraju, R.R., Cogswell, M., Das, A., Vedantam, R., Parikh, D., Batra, D. Grad-Cam: Visual explanations from deep networks via gradient-based localization. In *Proceedings of the IEEE International Conference on Computer Vision* 618–626 (2017).

[82] Attallah O (2023). RADIC: A tool for diagnosing COVID-19 from chest CT and X-ray scans using deep learning and quad-radiomics. Chemom. Intell. Lab. Syst..

[83] Mudrakola S, Hegde N (2023). Removal of noise on mammogram breast images using filtering methods. Concurr. Comput. Pract. Exp..

[84] Guarda L, Tapia J, Droguett EL, Ramos M (2022). A novel capsule neural network based model for drowsiness detection using electroencephalography signals. Expert Syst. Appl..

[85] Liu W, Li C, Rahaman MM, Jiang T, Sun H, Wu X, Hu W, Chen H, Sun C, Yao Y (2022). Is the aspect ratio of cells important in deep learning? a robust comparison of deep learning methods for multi-scale cytopathology cell image classification: From convolutional neural networks to visual transformers. Comput. Biol. Med..

